# Circulating cholesterol fuels SARS-CoV-2 replication via ORF3a

**DOI:** 10.1038/s44319-026-00907-0

**Published:** 2026-09-11

**Authors:** Jennifer M Hayashi, Taha Y Taha, Irene P Chen, Rahul K Suryawanshi, Danielle L Swaney, Maria McCavitt-Malvido, Mauricio Montano, Abdullah M Syed, Mir M Khalid, Camille R Simoneau, Pei-Yi Chen, Kristoffer E Leon, Chia-Lin Tsou, Erica Stevenson, Martin Gordon, Natalie R Chaplin, Danielle M Jorgens, Zhongde Wang, Andreas S Puschnik, Jennifer A Doudna, Nevan J Krogan, Melanie Ott

**Affiliations:** 1 J. David Gladstone Institutes, San Francisco, CA USA; 2 Department of Biology, Vassar College, Poughkeepsie, NY USA; 3 Department of Bioengineering and Therapeutic Sciences, University of California, San Francisco, San Francisco, CA USA; 4 Department of Medicine, University of California, San Francisco, San Francisco, CA USA; 5 Laboratory of Neurological Infections and Immunity, Division of Intramural Research, National Institute of Allergy and Infectious Diseases, Rocky Mountain Laboratories, NIH, Hamilton, MT USA; 6 Quantitative Biosciences Institute (QBI), University of California, San Francisco, San Francisco, CA USA; 7 Electron Microscope Laboratory, University of California, Berkeley, CA USA; 8 Department of Animal, Dairy, and Veterinary Sciences, Utah State University, Logan, UT USA; 9 Biohub, San Francisco, CA USA; 10 Department of Molecular and Cell Biology, University of California, Berkeley, Berkeley, CA USA; 11 Howard Hughes Medical Institute, University of California, Berkeley, Berkeley, CA USA; 12 Department of Chemistry, University of California, Berkeley, Berkeley, CA USA; 13 California Institute for Quantitative Biosciences (QB3), University of California, Berkeley, Berkeley, CA USA; 14 Molecular Biophysics and Integrated Bioimaging Division, Lawrence Berkeley National Laboratory, Berkeley, CA USA

**Keywords:** Membranes & Trafficking, Microbiology, Virology & Host Pathogen Interaction

## Abstract

High levels of “bad” cholesterol increase the risk of cardiovascular disease and are also linked to more severe COVID-19, though the reason has been unclear. We show that low-density lipoprotein (LDL) directly enhances SARS-CoV-2 RNA replication, especially in the Beta variant. The viral accessory protein ORF3a is both necessary and sufficient to increase cholesterol uptake in cell culture and hamster models. ORF3a stabilizes the host LDL receptor by displacing the PCSK9 chaperone, leading to greater cholesterol entry into cells. While wild-type ORF3a disrupts endosomal acidification and limits cholesterol recycling through interaction with VPS39 (part of the HOPS complex), the Beta variant does not, allowing more efficient cholesterol recovery and stronger LDL responsiveness. Overall, our findings show that SARS-CoV-2 can directly link its replication to host cholesterol levels via ORF3a. This mechanism may help guide antiviral strategies, particularly for individuals with high cholesterol.

## Synopsis

Viruses modify cellular membranes to generate replication organelles for protected viral factories. This study reveals that SARS-CoV-2 uses its accessory protein, ORF3a, to feed this demand for membranes by inducing cholesterol uptake via LDLR-mediated endocytosis.

- High extracellular cholesterol supports increased SARS-CoV-2 infection.
- SARS-CoV-2 increases LDL uptake via its ORF3a protein.
- Beta variant SARS-CoV-2 ORF3a optimizes LDL uptake and hydrolysis.

## Abstract (web summary)

Viruses modify cellular membranes to generate replication organelles for protected viral factories. This study reveals that SARS-CoV-2 uses its accessory protein, ORF3a, to feed this demand for membranes by inducing cholesterol uptake via LDLR-mediated endocytosis.

## Graphical abstract

**Figure:**
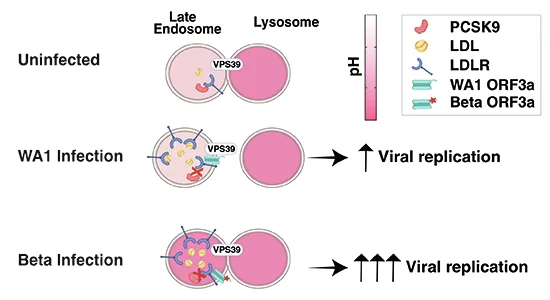

## Introduction

People with comorbidities like old age, obesity, and type 2 diabetes have an increased likelihood of severe COVID-19 disease and morbidity (Bhaskaran et al, 2021; Bonetti et al, 2003; Collaboration NCDRF 2016; Cummings et al, 2020; Gao et al, 2021; Hamer et al, 2020; Klop et al, 2013; Tamara and Tahapary, 2020). One reason might be hypercholesterolemia, as elevated levels of circulating cholesterol-rich low-density lipoprotein (LDL) correlate with worse patient outcomes. In support, individuals on statin treatment, which lowers circulating LDL, showed less severe disease (Daniels et al, 2020; Tan et al, 2020; Zhang et al, 2020), while individuals with a common genetic variant of apolipoprotein E4 (APOE4), which increases circulating LDL, experienced increased SARS-CoV-2 morbidity and mortality (Ostendorf et al, 2022). Fenofibrate, which induces lipoprotein lipase, has been shown to decrease virally induced lipid accumulation (Ehrlich et al, 2023) and viral entry (Davies et al, 2021). The same study used fenofibrate to treat patients infected with SARS-CoV-2, and they observed increased patient survival (Ehrlich et al, 2023). These clinical observations are replicated in hamster models of diet-induced obesity, which underscore the pathogenic role of LDL in SARS-CoV-2 infection (Ostendorf et al, 2022; Port et al, 2021; Safdari Lord et al, 2022). However, how hypercholesterolemia contributes to increased SARS-CoV-2 infection remains unknown.

In addition to the correlation between COVID-19 severity and circulating cholesterol, there is also evidence that SARS-CoV-2 infection depends on intracellular cholesterol levels. Multiple genome-wide CRISPR screens and viral-host interaction studies have identified intracellular cholesterol homeostasis as a key host pathway for SARS-CoV-2 infection (Gordon et al, 2020a; Gordon et al, 2020b; Hoffmann et al, 2021; Schneider et al, 2021; Stukalov et al, 2021; Wang et al, 2021a; Wei et al, 2021). Intracellular cholesterol supports the entry of several coronaviruses, such as severe acute respiratory syndrome coronavirus (SARS-CoV), Middle East respiratory syndrome coronavirus (MERS-CoV), and SARS-CoV-2 into cells (de Wilde et al, 2014; Li et al, 2007; Lu et al, 2008; Sanders et al, 2021; Wang et al, 2023). This dependence on intracellular cholesterol has been leveraged by therapeutic strategies using methyl-β-cyclodextrins, which bind and physically remove cholesterol from the plasma membrane (Sanders et al, 2021), or 25-hydroxycholesterol, which sequesters membrane cholesterol into intracellular lipid droplets (Abrams et al, 2020; Wang et al, 2020; Zang et al, 2020); both decreasing viral replication in cell culture models.

In most tissues, cholesterol homeostasis is maintained through exogenous lipoprotein uptake or de novo synthesis (Ikonen, 2008). But some tissues, including lungs, rely on circulating lipoprotein complexes to provide 80% of their cholesterol (Brown and Goldstein, 2009; Fessler and Summer, 2016; Gowdy and Fessler, 2013; Ikonen, 2008; Nistor and Simionescu, 1986; Turley et al, 1981). LDL takes up dietary cholesterol or cholesterol synthesized in the liver (Gotto, 1990; Grundy, 1983) and delivers it to tissues. Conversely, high-density lipoproteins (HDL) act as a recipient of excess intracellular cholesterol and shuttle it to the liver for excretion (Ikonen, 2008). Several observations suggest a link between cholesterol uptake via LDL and SARS-CoV-2 infection. First, protein-protein interaction studies have found that SARS-CoV-2 proteins interact with the LDL receptor (LDLR)  and with Neimann-Pick type C2 (NPC2), a chaperone that shuttles free cholesterol from the late endosome to the endoplasmic reticulum (ER) or the plasma membrane (Gordon et al, 2020b; Stukalov et al, 2021). Second, genome-wide CRISPR screens showed that LDLR, NPC1, and an acid hydrolase (LIPA) were important host factors for SARS-CoV-2 infection (Daniloski et al, 2021; Schneider et al, 2021; Wang et al, 2021a).

Given the lung’s reliance on LDL uptake for cholesterol homeostasis, and the fact that obese patients, who have increased circulating LDL, are highly susceptible to severe COVID-19, we hypothesize that LDL uptake by lung cells facilitates SARS-CoV-2 infection. Previous studies have focused on how the depletion of cellular cholesterol decreased infection, but not on the mechanism by which the virus uses cholesterol during infection. Critically, how extracellular LDL levels affect viral infection and whether the virus changes intracellular cholesterol metabolism remain unknown. Here, we use LDL uptake assays to demonstrate that the virus benefits from increased extracellular cholesterol at the genomic replication step. Subsequently, we show that the viral protein ORF3a increases LDL uptake, likely through its interaction with LDLR. ORF3a is a putative channel protein normally associated with deacidification of endosomes (Kern et al, 2021; Miao et al, 2021; Michelucci et al, 2025; Zhang et al, 2022a; Zhang et al, 2021). We demonstrate that specific point mutations in ORF3a, associated with increased viral pathogenesis (Nagy et al, 2021) and found in the Beta Variant (i.e., S171L), not only increased LDL uptake, but also endosomal acidification. We propose that SARS-CoV-2 ORF3a induces LDL uptake to compensate for the inefficient LDL hydrolysis resulting from ORF3a-induced deacidification. Overall, our findings may explain the high prevalence of severe COVID-19 among individuals with high levels of circulating cholesterol.

## Results

### Extracellular LDL enhances infection by pathogenic SARS-CoV-2 variants

**Figure 1:**
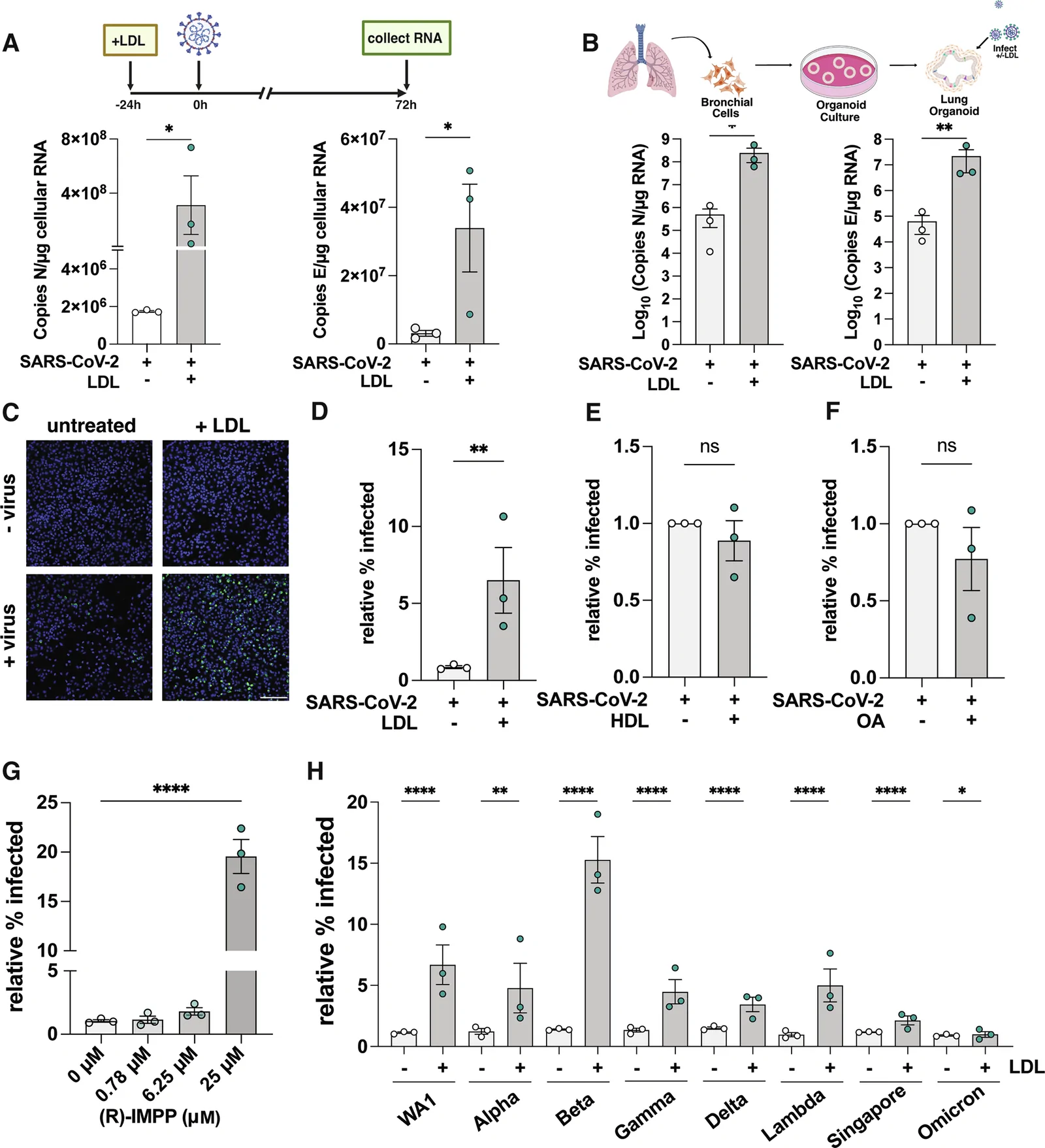
Extracellular LDL enhances SARS-CoV-2 infection of human airway cells. RT-qPCR of cellular RNA from A549-ACE2 cells (**A**) or human airway organoids (**B**) infected with SARS-CoV-2 WA1 in the presence or absence of LDL using primers against viral nucleocapsid (N, left) and envelope (E, right). *N* = 3 biological replicates. Unpaired *t* test. Fluorescence microscopy (**C**) and quantification (**D**) of dsRNA-positive (green) A549-ACE2 cells infected with SARS-CoV-2 WA1 in the presence or absence of LDL. Scale bar = 500 µm. *N* = 3 biological replicates. Unpaired *t* test. (**E**) Quantification of dsRNA-positive A549-ACE2 cells infected with SARS-CoV-2 WA1 in the presence or absence of high-density lipoproteins (HDL, **E**) or oleic acid (OA, **F**). *N* = 3 biological replicates. Unpaired *t* test. (**G**) Quantification of dsRNA-positive cells treated with (R)-IMPP compared to untreated controls. *N* = 3 biological replicates. One-way ANOVA. (**H**) Quantification of dsRNA-positive A549-ACE2 cells infected with SARS-CoV-2 WA1, Alpha, Beta, Gamma, Delta, Lambda, Singapore (GSAID: EPI_ISL_414378) (Su et al, 2020), and Omicron variants treated with or without LDL. Infections were performed at an MOI of 0.1 for 72 h. *N* = 3 biological replicates. Unpaired *t* test. Data information: In (**A**, **B**, **D**–**H**), data are presented as mean ± SEM. ****P < 0.0001, ***P < 0.001, **P < 0.01, *P < 0.05. Source data are available online for this figure.

**Figure EV1:**
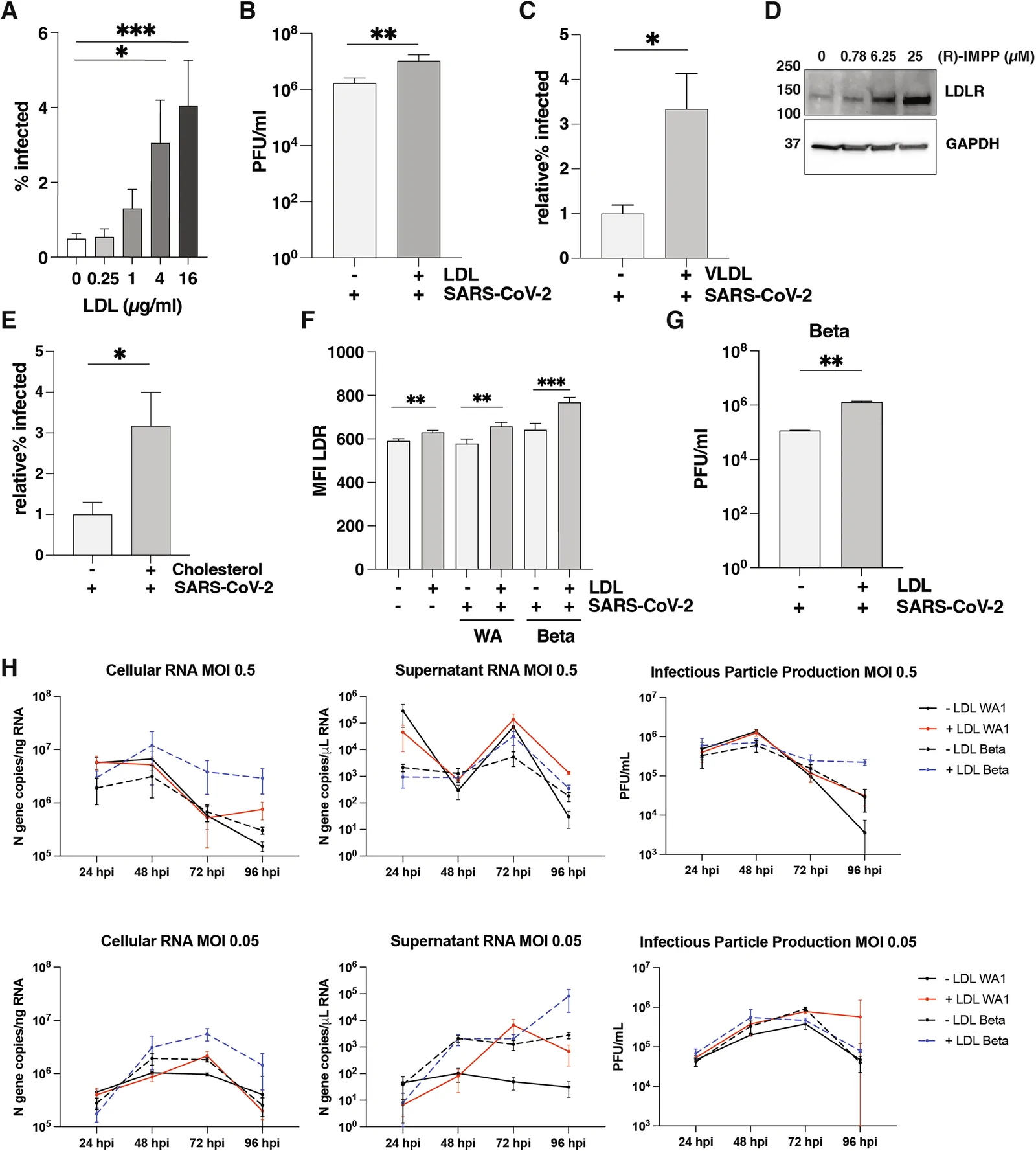
SARS-CoV-2 infection of human airway cells is enhanced by extracellular LDL. (**A**) Dose-dependent increase in SARS-CoV-2 infection in A549-ACE2 cells upon LDL treatment, measured by quantifying dsRNA-positive cells via automated imaging. *N* = 3 biological replicates. One-way ANOVA. (**B**) Infectious virion production from A549-ACE2 cells following LDL treatment measured by plaque forming units (PFU) in Vero E6 cells. *N* = 3 biological replicates. Paired *t* test. (**C**) Quantification of dsRNA-positive A549-ACE2 cells infected with SARS-CoV-2 WA1 in the presence or absence of VLDL. *N* = 3 biological replicates. Unpaired *t* test. (**D**) Representative Western blot of LDLR and GAPDH expression in A549-ACE2 cells treated with increasing doses of (R)-IMPP for 24 h. (**E**) Quantification of dsRNA-positive A549-ACE2 cells treated with 40 µM cholesterol complexed with methyl-beta-cyclodextrin. *N* = 3 biological replicates. Unpaired *t* test. (**F**) Quantification of mean fluorescent intensity (MFI) of LipidTOX Deep Red (LDR) in A549-ACE2 cells infected with WA1 or Beta variant SARS-CoV-2, with or without LDL treatment. MOI 0.1 for 72 h. *N* = 3 biological replicates. Unpaired *t* test. (**G**) Beta variant infectious virion production from A549-ACE2 cells upon LDL treatment measured by PFU in Vero E6 cells. *N* = 3 biological replicates. Unpaired *t* test. (**H**) Copies of viral RNA (cellular and supernatant) measured by RT-qPCR against the viral N gene, and infectious virion production measured by PFU in A549-ACE2 cells over 4 days. *N* = 3 biological replicates. Unpaired *t* test. Data information: In (**A**–**C**, **E**–**H**), data are presented as mean ± SEM. ***P < 0.001, **P < 0.01, *P < 0.05.

To test whether SARS-CoV-2 infection directly benefits from extracellular cholesterol, we incubated A549 lung epithelial cells overexpressing the ACE2 receptor (A549-ACE2) or primary human airway organoids (HAOs) with commercially available, serum-isolated lipids—LDL, HDL, very low-density lipoprotein (VLDL), or oleic acid, the main precursor for triglycerides—before infecting them with SARS-CoV-2 USA-WA1/2020 (WT) (Fig. 1). The addition of LDL increased viral RNA production in A549-ACE2 cells (Fig. 1A) and in HAOs (Fig. 1B). LDL and VLDL treatment, but not HDL or oleic acid treatment, increased the number of cells with viral RNA and enhanced the overall signal for viral RNA replication across cells (Figs. 1C–F and EV1A–C). At the concentration used, oleic acid induces triglyceride synthesis, without inducing lipid droplet storage, and does not increase infection. As HDL is primarily a recipient cholesterol complex that provides cholesterol via a low-throughput “nibbling” mechanism, these findings suggest that the uptake of cholesterol, rather than its removal, is important for viral infection. To test this hypothesis, we inhibited proprotein convertase subtilisin/kexin type 9 (PCSK9), a protein that targets LDLR for degradation, by treating cells with the small molecule inhibitor (R)-IMPP (Petersen et al, 2016). PCSK9 inhibition increased LDLR abundance as expected and enhanced SARS-CoV-2 infection (Figs. 1G and EV1D). Supporting the importance of the cholesterol moiety of LDL, cells treated with purified cholesterol carried by methyl-beta cyclodextrins also showed an increase in SARS-CoV-2 infection (Fig. EV1E). Together, these results demonstrate that SARS-CoV-2 preferentially exploits cholesterol uptake over other lipids to promote infection.

Interestingly, LDL increased infection across all variants of SARS-CoV-2 tested (Fig. 1H). Notably, the Beta variant showed the strongest enhancement in infection, viral particle production, lipid droplet accumulation, and viral RNA accumulation compared to WA1 (Figs. 1H and EV1F–H). During its circulation, the Beta variant caused greater mortality than the ancestral WA1 and Alpha strains (Abu-Raddad et al, 2022; Cohen et al, 2022; Radvak et al, 2021; Tarres-Freixas et al, 2022). In contrast, the Omicron variant showed less infection enhancement in response to LDL (Fig. 1H). Our findings suggest a possible strategy employed by the Beta variant to increase its own replication.

### SARS-CoV-2 RNA replication is boosted by exogenous LDL

The increased pathogenicity of the Beta variant is in part due to its Spike protein, which increases syncytia formation, and hence viral entry (McGrath et al, 2022; Rajah et al, 2021). However, a chimeric virus carrying the Beta Spike in a WT background failed to recapitulate the pathogenic effects of the Beta variant in a mouse model, suggesting that viral proteins other than Spike also contribute to its pathogenicity (McGrath et al, 2022; Rajah et al, 2021). The Beta variant differs from the WT strain at 18 sites: five in ORF1ab, two in ORF3a, and one each in envelope, ORF8, and nucleocapsid, in addition to eight in Spike. We hypothesized that its increased sensitivity to LDL was due to one or more of the ten mutations outside Spike.

**Figure 2:**
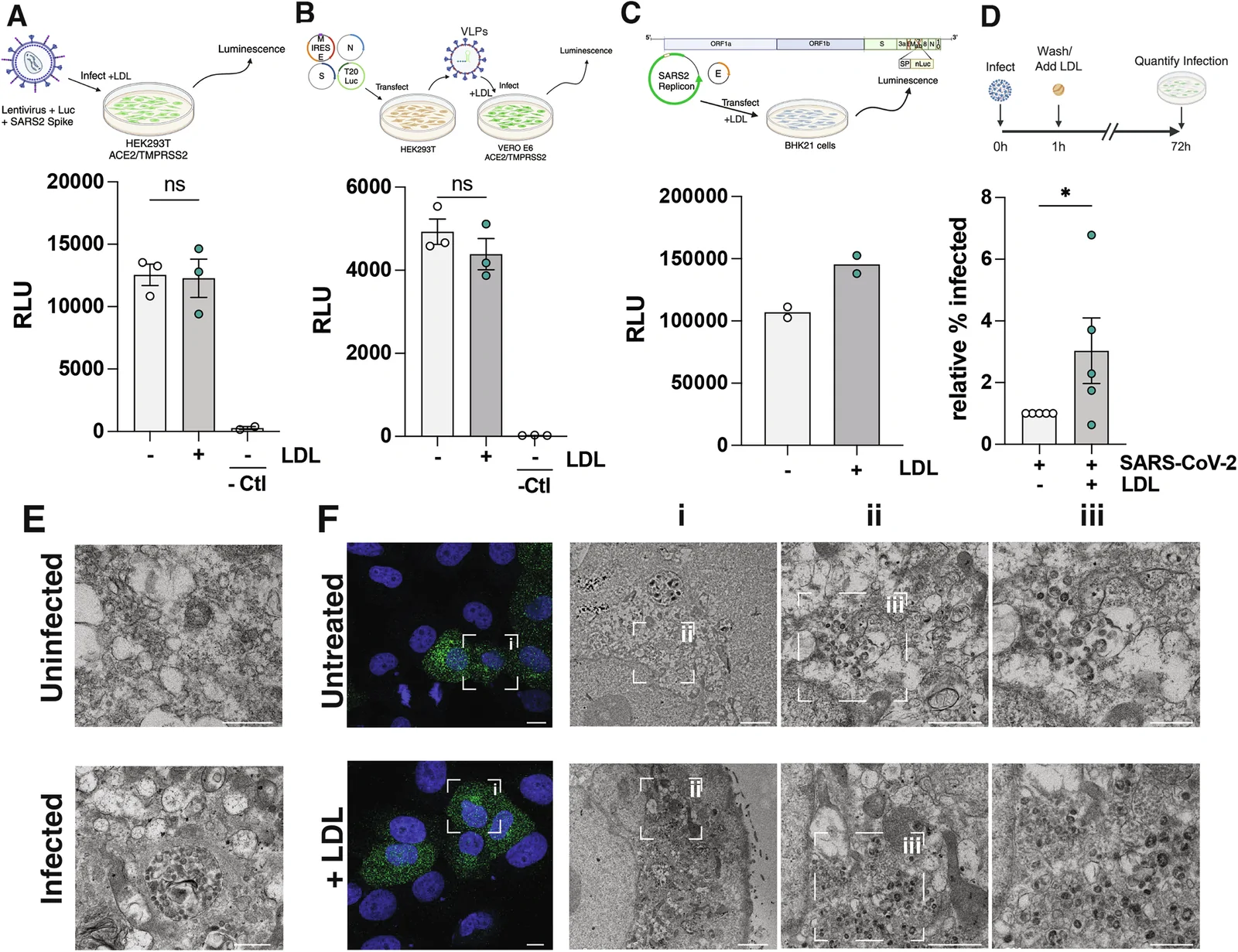
LDL addition enhances viral RNA replication. (**A**) Luminescence of HEK293T-ACE2/TMPRSS2 cells after transduction with Spike-carrying pseudotyped lentiviral vectors with and without LDL. Negative control is supernatant from transfections without the spike plasmid. *N* = 3 biological replicates. Unpaired *t* test. (**B**) Luminescence of HEK293T-ACE2/TMPRSS2 cells after transduction with virus-like particles produced in HEK293T cells with and without LDL. Negative control is supernatant from transfections without the spike plasmid. *N* = 3 biological replicates. Unpaired *t* test. (**C**) Luminescence of BHK21 cells transfected with SARS-CoV-2 WA1 replicon in the presence or absence of LDL. *N* = 2 biological replicates. (**D**) Quantification of dsRNA-positive A549-ACE2 cells infected with SARS-CoV-2 WA1 for 1 h, washed, and replaced with media with or without LDL. *N* = 5 biological replicates. Unpaired *t* test. (**E**) Representative TEM images of membranous structures in VERO-ACE2/TMPRSS2 cells with or without SARS-CoV-2 infection. Scale bar = 500 nm. (**F**) Representative images of correlative light and electron microscopy (i, ii, iii) of VERO-ACE2/TMPRSSS2 cells infected with mNeonGreen single-round WA1 virus. Scale bars = 10 µm, 2 µm, 1000 nm, and 500 nm, respectively. Data information: In (**A**–**D**), data are presented as mean ± SEM. **P < 0.01, *P < 0.05. Source data are available online for this figure.

**Figure EV2:**
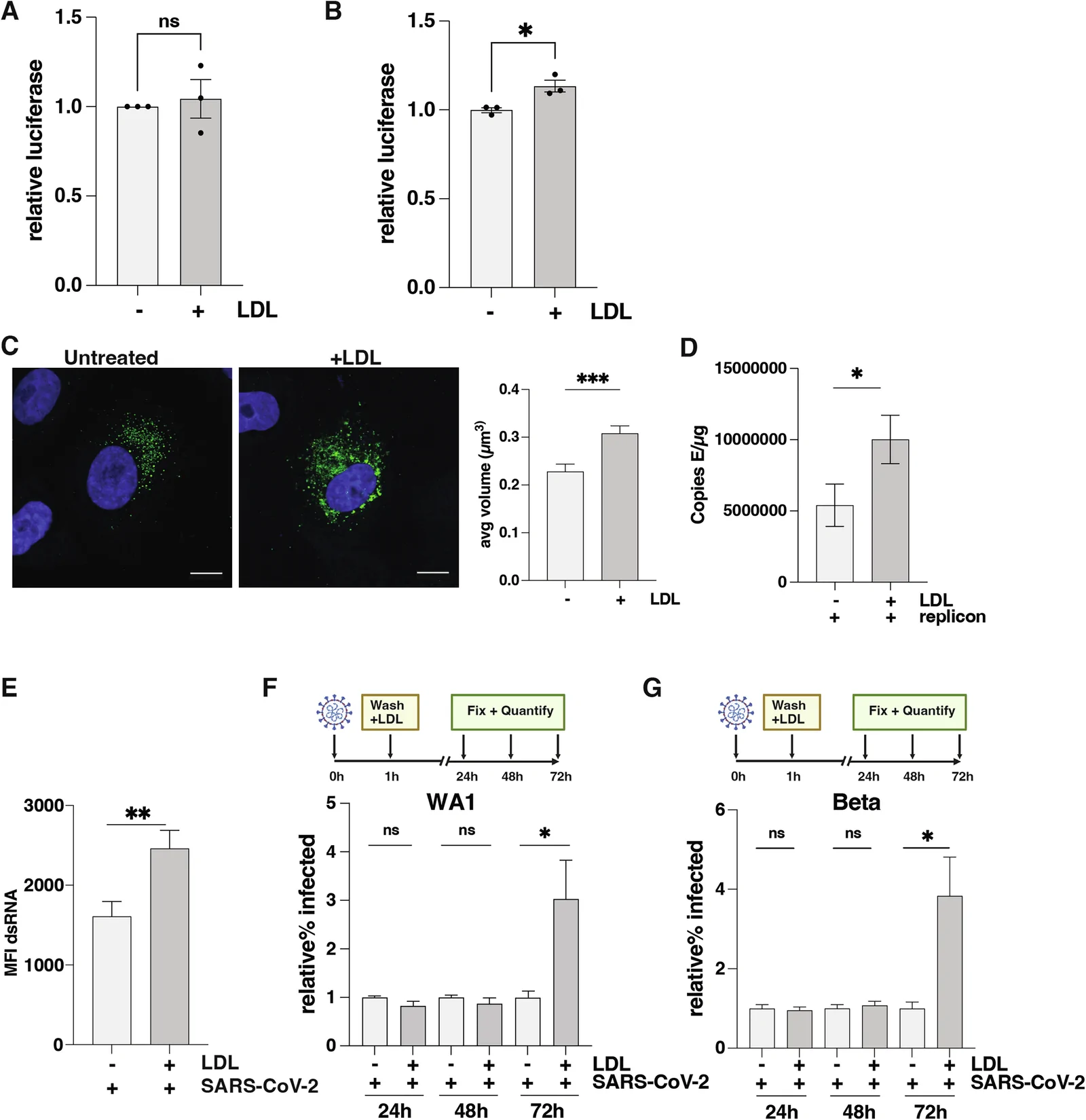
LDL treatment increases viral dsRNA-positive complexes. (**A**) Luminescence analysis of A549-ACE2 cells transduced with Spike-carrying pseudotyped lentiviral vectors with or without LDL. *N* = 3 biological replicates. Unpaired *t* test. (**B**) Luminescence analysis of A549-ACE2 cells infected with single-round SARS-CoV-2 WA1 replicon in the presence or absence of LDL. *N* = 3 biological replicates. Unpaired *t* test. (**C**) Representative images and quantification of dsRNA complex volume in Vero E6-ACE2/TMPRSS2 cells infected with single-round SARS-CoV-2 WA1 replicon virus in the presence or absence of LDL, stained for dsRNA (green) replication complex volume. Scale bar = 10 µm. *N* = 3 biological replicates. Unpaired *t* test. (**D**) RT-qPCR analysis of viral E gene from cellular RNA in A549-ACE2 cells infected with single-round replicon of SARS-CoV-2 WA1 in the presence or absence of LDL. *N* = 3 biological replicates. Unpaired *t* test. (**E**) Quantification of dsRNA fluorescence mean intensity (MFI) in A549-ACE2 cells infected with SARS-CoV-2 WA1 for 1 h, washed and replaced with media with or without LDL. *N* = 3 biological replicates. Unpaired *t* test. Quantification of dsRNA-positive A549-ACE2 cells infected with SARS-CoV-2 WA1 (**F**) or SARS-CoV-2 Beta (**G**) for 1 h, washed, and replaced with media with or without LDL at different time points. *N* = 3 biological replicates. Unpaired *t* test. Data information: In (**A**–**D**), data are presented as mean ± SEM. ***P < 0.001, **P < 0.01, *P < 0.05.

To determine at which step of the viral life cycle LDL benefits infection, we used several subgenomic infection systems. To measure viral entry, we used pseudotyped viruses (PTV), which are replication-defective lentiviral particles that express the SARS-CoV-2 Spike protein and deliver luciferase RNA to recipient cells through the ACE2 receptor (Deng et al, 2021). LDL exposure did not increase the entry of these PTVs into target cells that expressed only ACE2 (A549-ACE2) or cells that expressed ACE2 along with the transmembrane protease, serine 2 (TMPRSS2) enzyme necessary for Spike priming (HEK293T-ACE2/TMPRSS2 cells) (Figs. 2A and EV2A). This finding contrasts with previous studies that primarily focused on viral entry (Wang et al, 2023), which found that cholesterol-rich lipoproteins increase viral infection. Experimental setups with different lipoproteins, cell models, and viral systems may explain the differences in our observations. Then, we used virus-like particles (VLP) (Syed et al, 2021), which express all viral structural proteins and model the assembly and egress of authentic SARS-CoV-2 particles. LDL treatment did not affect VLP production, as measured by comparable delivery of luciferase RNA into target cells (Fig. 2B). In both systems, spike expression was required for luciferase signal as bald (without spike) particles were unable to deliver cargo into target cells (- Ctl).

Finally, to measure viral genomic replication, we used a replicon system for SARS-CoV-2 in which the envelope (E) protein is deleted and the accessory proteins ORF7a and ORF7b are replaced with a secreted nanoluciferase (Taha et al, 2023). Treatment with LDL increased the nanoluciferase signal in the cultures transfected with the replicon, indicating that RNA replication was stimulated by LDL treatment independent of viral entry or assembly (Figs. 2C and EV2B). In support of this, replicon-infected, LDL-treated cells contained over larger double-stranded RNA (dsRNA; green) complexes (Fig. EV2C) and more viral RNA compared to untreated, infected cells (Fig. EV2D). Additionally, LDL-treated, WT virus-infected A549-ACE2 cells had increased dsRNA staining compared to untreated cells, illustrating increased viral replication complexes and reinforcing our replicon data (Fig. EV2E). A previous study demonstrated the reorganization and colocalization of Filipin and dsRNA staining during HCov-229E infection (Trimarco et al, 2021), suggesting that coronaviral RNA replication requires increased cholesterol. We confirmed that LDL affected a post-entry step by treating A549-ACE2 cells with LDL after they had been exposed to infectious virus for 1 h. Post-infection LDL treatment led to a 3-fold increase in viral replication for the WA1 strain and a fourfold increase for the Beta strain after 72 h, supporting the model that LDL promotes sustained viral replication (Figs. 2D and EV2F,G).

Finally, to examine ultrastructural changes during viral replication, we examined intracellular membranes that function as hubs for viral RNA replication using correlative light and electron microscopy (CLEM). SARS-CoV-2 replication occurs in the double membrane vesicles (DMVs) (Knoops et al, 2008) and causes substantial reorganization of host intracellular membranes (Fig. 2E). We observed an increased abundance and density of DMVs, and an increased number of small open double-membrane spherules, and assembling virions (Maier et al, 2013; Snijder et al, 2020) in the infected, LDL-treated cells compared to untreated controls (Fig. 2F). Double membrane spherules remain elusive (Maier et al, 2013; Snijder et al, 2020). The enrichment of these membranous structures during LDL treatment supports the model in which LDL-derived cholesterol during infection enhances the formation of intracellular membranes important for viral RNA replication.

### SARS-CoV-2 infection and expression of ORF3a alone enhance LDL uptake

**Figure 3:**
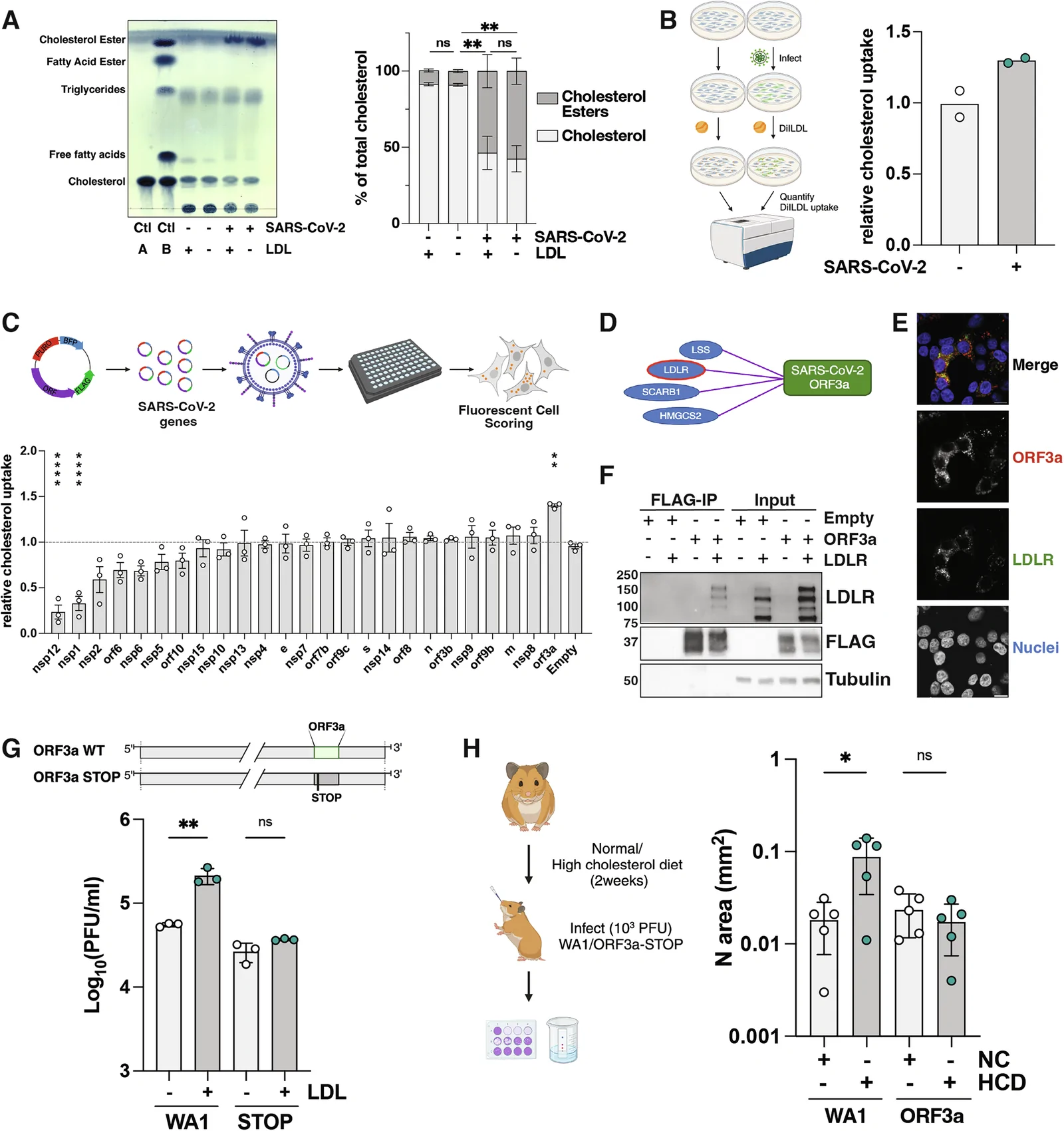
SARS-CoV-2 enhances LDL uptake and interacts with LDLR. (**A**) Thin-layer chromatography and quantification of lipids extracted from uninfected and infected A549-ACE2 cells (WA1 MOI 0.1 for 72 h). *N* = 3 biological replicates. One-way ANOVA. (**B**) DiI-LDL uptake in A549-ACE2 cells infected with SARS-CoV-2 WA1. *N* = 2 biological replicates. (**C**) DiI-LDL uptake in A549 cells expressing SARS-CoV-2 proteins relative to empty vector control. *N* = 3 biological replicates. One-way ANOVA. (**D**) ORF3a interactome with cholesterol-related proteins (Stukalov et al, 2021). (**E**) Confocal microscopy of ORF3a-FLAG (red) and GFP-LDLR (green) -expressing HEK293T cells. Scale bar = 10 µm. (**F**) Co-immunoprecipitation by anti-FLAG beads of ORF3a-FLAG with GFP-LDLR expressed in HEK293T cells. (**G**) Infectious virion production (PFU/ml) from SARS-CoV-2 WA1 and ORF3a-STOP infectious clones generated in Calu-3 cells after 48 h in the presence or absence of LDL. *N* = 3 biological replicates. Unpaired *t* test. (**H**) Golden Syrian Hamsters were preconditioned with a high cholesterol diet (HCD) or normal diet (ND) for 2 weeks prior to infection, infected with 10^3^ PFU SARS-CoV-2 WA1 or ORF3a-STOP infectious clones for 48 h. Immunohistochemistry was performed on lung sections to quantify area of antibody staining against the SARS-CoV-2 nucleocapsid protein. *N* = 5 animals per group. One-way ANOVA. Data information: In (**A**–**C**, **G**, **H**), data are presented as mean ±  SEM. ****P < 0.0001, ***P < 0.001, **P < 0.01, *P < 0.05. Source data are available online for this figure.

**Figure EV3:**
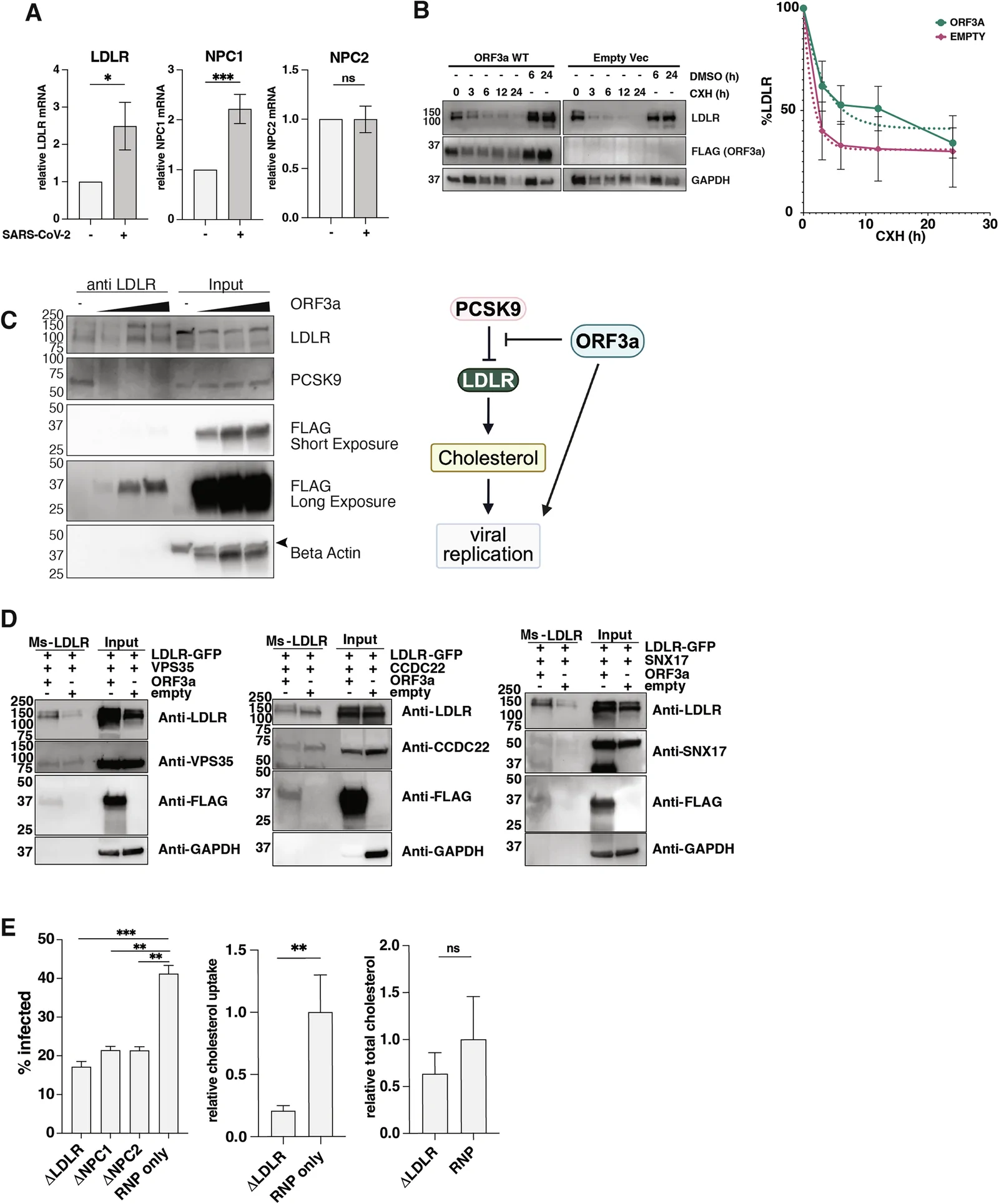
SARS-CoV-2 requires cholesterol uptake genes. (**A**) RT-qPCR of cellular RNA from A549-ACE2 cells infected with SARS-CoV-2 WA1 using primers against human *ldlr*, *npc1*, and *npc2*. *N* = 3 biological replicates. Unpaired *t* test. (**B**) Representative Western blots and quantification of cycloheximide (CXH) or DMSO-treated HEK293T cells expressing ORF3a-FLAG and GFP-LDLR over time. *N* = 3 biological replicates. Dotted lines indicate nonlinear regression best-fit curves. *R*^2^ = 0.7047 (ORF3a) and 0.6684 (empty vector control). (**C**) Model and representative Western blot of anti-LDLR co-immunoprecipitation of LDLR, PCSK9, ORF3a-FLAG, and β-actin in HEK293T cells expressing ORF3a-FLAG. Arrowhead indicate expected band for β-actin. (**D**) Representative western blot of anti-LDLR co-immunoprecipitation of LDLR, VPS35, CCDC22, SNX17, ORF3a-FLAG, and GAPDH in HEK293T cells overexpressing GFP-LDLR, ORF3a-FLAG, and respective CCC/WASH/HOPS complex members. (**E**) Left panel, Quantification of dsRNA-positive A549-ACE2 cells infected with SARS-CoV-2 WA1 in CRISPR knockout lines (∆LDLR, *∆*NPC1, or ∆NPC2) or RNP only controls. *N* = 3 biological replicates. Unpaired *t* test. Middle panel, DiI-LDL uptake by mean fluorescence intensity in ∆LDLR or RNP only control A549-ACE2 cells. *N* = 3 biological replicates. Unpaired *t* test. Right panel, total intracellular cholesterol using Filipin III staining (mean fluorescence intensity) compared to RNP control in ∆LDLR or RNP only A549-ACE2 cells. *N* = 3 biological replicates. Unpaired *t* test. Infections performed at an MOI of 0.1 for 72 h. Data information: In (**A**, **B**, **E**), data are presented as mean ±  SEM. ***P < 0.001, **P < 0.01, *P < 0.05.

Our data demonstrate that LDL and cholesterol enhance SARS-CoV-2 infection, we next examined whether SARS-CoV-2 infection, with or without LDL addition, altered other lipids of A549-ACE2 cells using thin-layer chromatography (TLC). Strikingly, esterified cholesterols were enriched in infected cells compared to uninfected controls, regardless of LDL treatment (Fig. 3A). Cholesterol esters are the more stable storage form of cholesterol, found in lipid droplets and transported in LDL particles. To determine if this enrichment is caused by enhanced cholesterol uptake, we treated cells with fluorescently labeled LDL (DiI-LDL) and measured intracellular fluorescence. Infection of A549-ACE2 cells increased LDL uptake by ~20% relative to uninfected controls, supporting the model that viral infection optimizes LDL uptake for infection enhancement (Fig. 3B). Consistent with this model, infected cells expressed higher levels of key cholesterol uptake genes, such as the LDLR and NPC1, suggesting that viral replication requires replenishment of cellular cholesterol through LDL uptake (Fig. EV3A). Small molecule inhibition of oxysterol-binding protein (OSBP) prevents movement of cholesterol between the ER and Golgi apparatus, a function required for normal cholesterol homeostasis, and lowers coronavirus replication (Ma-Lauer et al, 2024). Combined with our data, this suggests that LDL uptake maximizes infection by maintaining viral replication organelles.

To identify the viral factor responsible for enhanced LDL uptake, we expressed SARS-CoV-2 proteins individually in A549 cells and measured the relative rate of DiI-LDL uptake (Fig. 3C). While some proteins significantly decrease DiI-LDL uptake, SARS-CoV-2 ORF3a expression alone increased LDL uptake by over 30%, similar to what was observed during viral infection (Fig. 3B,C). SARS-CoV-2 ORF3a is a small glycosylated transmembrane protein that forms a homo-tetrameric ion channel, which inhibits the fusion of late endosomes and lysosomes, preventing their acidification, and assisting in viral egress (Bignon et al, 2022; Chen et al, 2021; Ghosh et al, 2020; Hayn et al, 2021; Kern et al, 2021; Miao et al, 2021; Michelucci et al, 2025; Oostra et al, 2006; Solvik et al, 2022; Zhang et al, 2022a). It was determined that ORF3a interferes with the GTP hydrolysis cycle of Rab7, which benefits virion production (Walia et al, 2024). Additionally, ORF3a is a major viral pathogenesis factor as it downregulates MHC-I and inhibits JAK/STAT signaling to dampen the antiviral response (Arshad et al, 2023; Ghosh et al, 2020; Miao et al, 2021; Wang et al, 2021b; Xu et al, 2022; Zhang et al, 2021). Most notably, viruses with an *orf3a* deletion cause lower mortality in animal models (Liu et al, 2022). Our data show a new function of ORF3a–enhanced LDL uptake–that may be linked to its role in pathogenesis.

### ORF3a interacts with mature LDLR in endosomes

In an unbiased affinity purification-mass spectrometry (AP-MS) study, ORF3a was shown to bind to a number of host proteins, including the LDLR (Stukalov et al, 2021) (Fig. 3D). We confirmed this interaction by co-expressing FLAG-tagged ORF3a and GFP-tagged LDLR in HEK293T cells. LDLR internalizes with LDL through clathrin-mediated endocytosis, where dissociation and cholesterol recovery are induced by acidification of the endosomal lumen (Davis et al, 1987; Vos and van de Sluis, 2021). Indeed, confocal microscopy confirmed that SARS-CoV-2 ORF3a and host LDLR colocalized at intracellular endosomes (Fig. 3E). After immunoprecipitation, we identified LDLR as a bona fide ORF3a-interacting protein (Fig. 3F). Notably, expression of ORF3a alone induced the appearance of higher molecular bands for LDLR, indicating the accumulation of the mature receptor (Cummings et al, 1983; Davis et al, 1986; Ishibashi et al, 1993; Kulseth et al, 2010; Li et al, 2004; Maxwell et al, 2005; Tveten et al, 2013). This mature form of LDLR was specifically co-immunoprecipitated by ORF3a, suggesting that ORF3a is not interacting with newly synthesized LDLR in the ER, but rather induces the accumulation of mature LDLR in the cell. In support of this model, we found that expression of ORF3a prevented binding of LDLR to PCSK9, a chaperone targeting mature LDLR for degradation (Maxwell et al, 2005), thus enhancing its half-life in cells expressing ORF3a (Fig. EV3B,C).

In contrast, interactions of LDLR with components of the COMMD/CCDC22/CCDC93 (CCC) and Wiskott-Aldrich syndrome protein and SCAR homologue (WASH) complexes, involved in the recycling of internalized LDLR and other receptors back to the cell surface (Bartuzi et al, 2016), were not altered by ORF3a expression (Fig. EV3D). We confirmed that LDLR is a key component of the viral life cycle by CRISPR-Cas9-mediated knock-out of LDLR, NPC1, and NPC2 in A549-ACE2 cells, which decreased viral infections (Fig. EV3E).

### ORF3a is required for effective SARS-CoV-2 replication in vitro and in vivo

To test the role of ORF3a in mediating LDL’s effects on infection, we generated an infectious clone (Taha et al, 2023) of the ancestral WA1 strain of SARS-CoV-2 harboring a mutation to abrogate ORF3a expression. Unlike previous studies, which delete the entire *orf3a* RNA (Liu et al, 2022), we introduced a premature stop codon at the first amino acid position (M*), blocking ORF3a translation, while minimizing potential changes to viral genomic structure. When the mutant and WT virus-infected A549-ACE2 cells were pretreated with LDL, we noted a decrease in LDL responsiveness in the absence of ORF3a expression, confirming the critical role of ORF3a in LDL-mediated enhancement of viral infection (Fig. 3G).

**Figure EV4:**
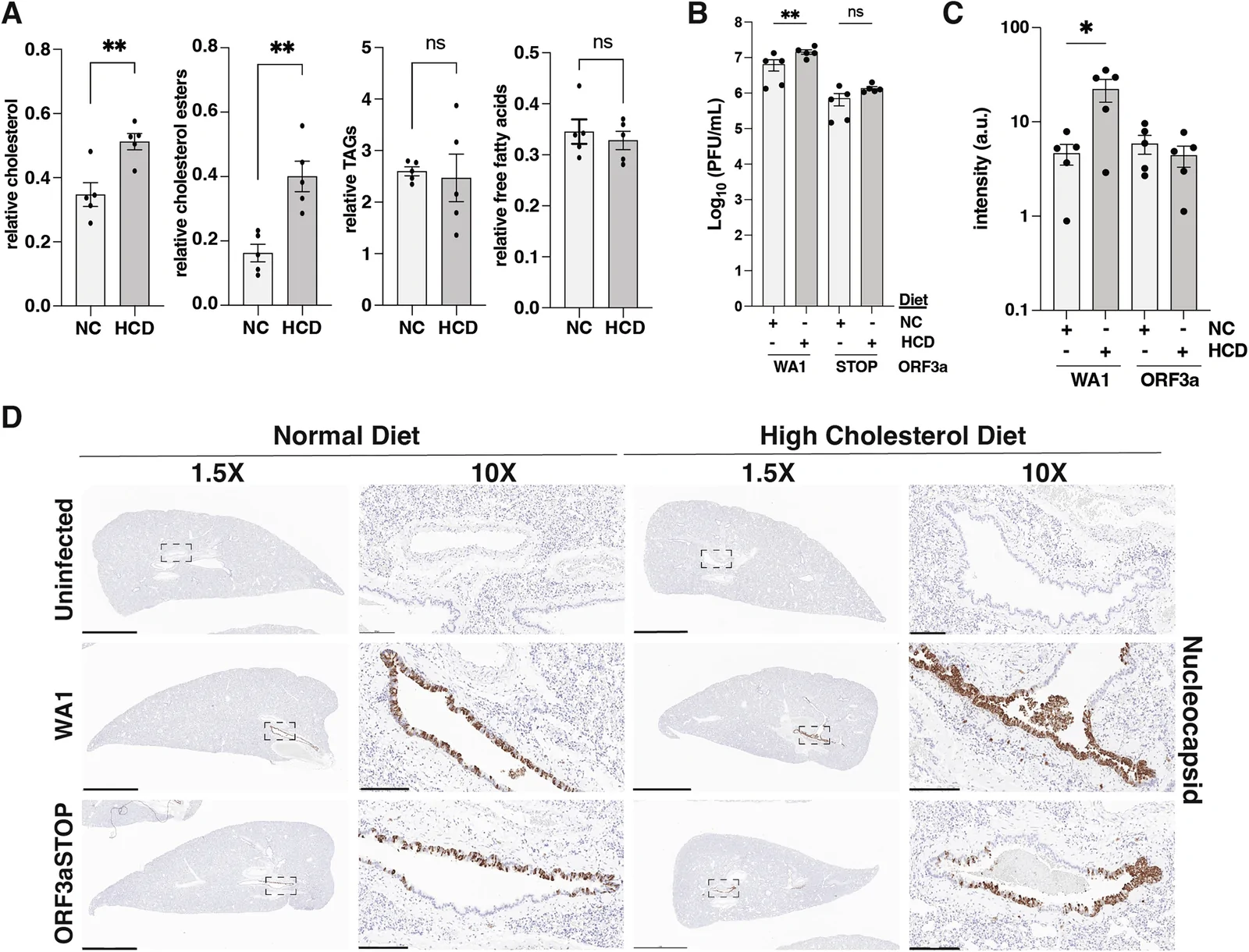
SARS-CoV-2 infection is enhanced in vivo with a high cholesterol diet. (**A**) Quantification of relative spot intensities of cholesterol, cholesterol esters, triglycerides, and free fatty acids from TLCs (normalized to 100 µg lipid standards) from uninfected lungs of Golden Syrian hamsters fed normal chow (NC) or high cholesterol diet (HCD). *N* = 5 animals per group. One-way ANOVA. (**B**) Infectious virion production in upper airway tissue from infected NC- or HCD-fed Golden Syrian Hamsters. *N* = 5 animals per group. One-way ANOVA. (**C**) Quantification of SARS-CoV-2 nucleocapsid staining in lungs by immunohistochemistry, shown as integrated density. *N* = 5 animals per group. One-way ANOVA. (**D**) Representative immunohistochemistry images of uninfected, WA1-infected, and ORF3a-STOP-infected hamster lungs, stained against viral N protein at ×1.5 zoom (left panel, scale bar = 2 mm) and 10X zoom (right panel, scale bar = 200 µm), fed either NC or HCD. Data information: In (**A**–**C**), data are presented as mean ± SEM. **P < 0.01, *P < 0.05.

To examine this effect in vivo, we infected Golden Syrian hamsters (*Mesocricetus auratus*), which were preconditioned with either a normal or a high cholesterol (0.5% cholesterol and 15% lard) diet for 2 weeks prior to infection with WT and ORF3a-STOP infectious clones (Figs. 3H and EV4A). WT-infected animals responded to the high cholesterol diet with a significant increase in viral infection and replication, while animals infected with the ORF3a-STOP virus did not, confirming the critical role of ORF3a and circulating cholesterol in enhanced viral replication in vivo (Figs. 3H and EV4B–D). Notably, the ORF3a-STOP virus propagated less efficiently in animals on a normal cholesterol diet, underscoring ORF3a’s critical role in viral infection (Fig. EV4B) (Zhang et al, 2021).

### ORF3a from the Beta variant changes the acidification of LDL-positive endosomes

**Figure 4:**
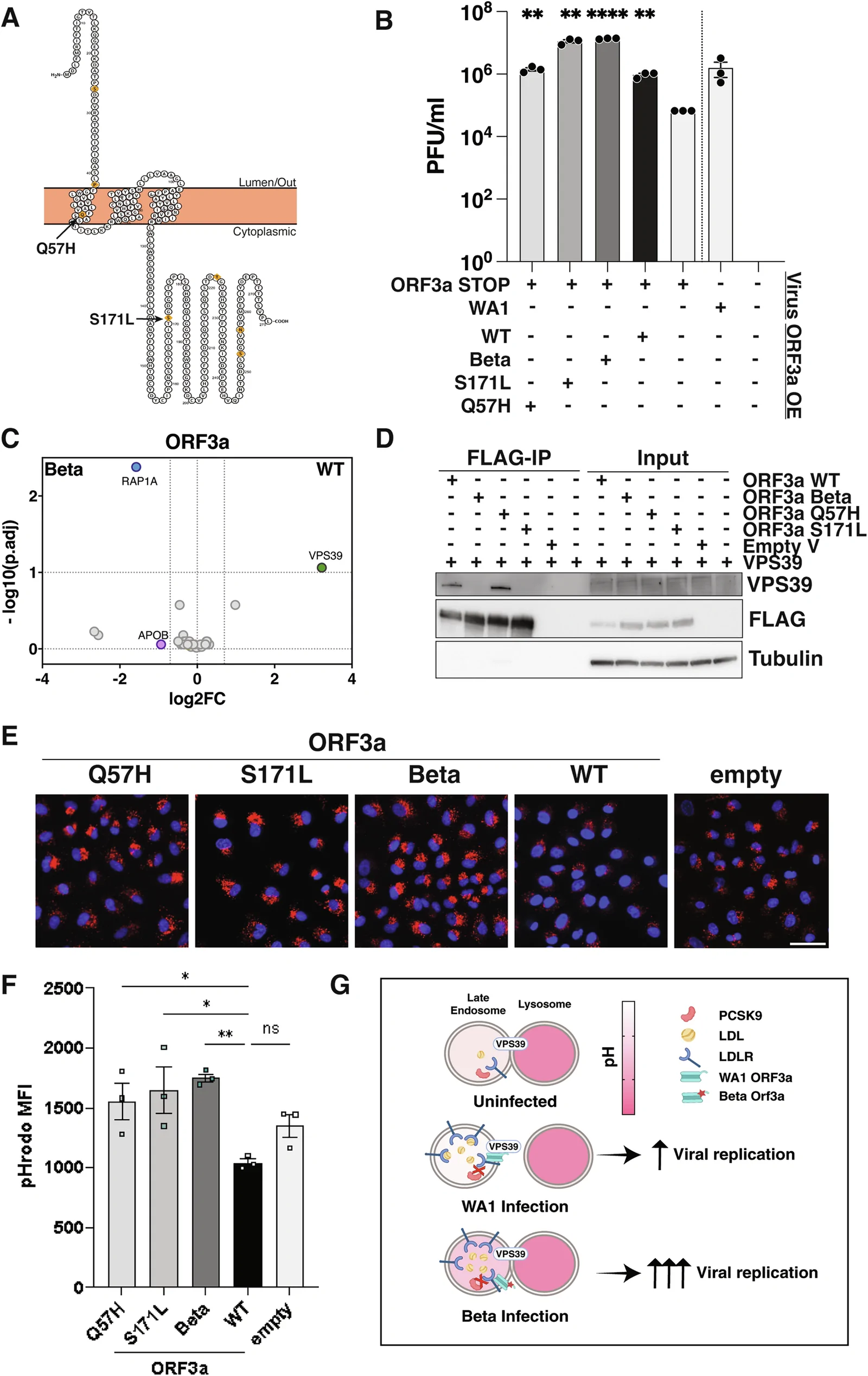
SARS-CoV-2 WT carrying Beta variant ORF3a mutations is more sensitive to LDL augmentation. (**A**) Predicted structure of ORF3a generated using Protter (Omasits et al, 2014); Beta variant mutations are marked with arrows. (**B**) Infectious virion production (PFU/ml) from SARS-CoV-2 ORF3a-STOP and WA1 infectious clones at MOI 0.01, generated in Calu-3 cells overexpressing different genotypes of ORF3a after 48 h. *N* = 3 biological replicates. One-way ANOVA. (**C**) Log_2_ fold change in abundance of significantly enriched or depleted host proteins from affinity purified-mass spectrometry of HEK293T cells overexpressing WT or Beta ORF3a-FLAG. *N* = 3 biological replicates. (**D**) Anti-FLAG co-immunoprecipitation of HEK293T cells transfected with different ORF3a-FLAGs, GFP-LDLR, and VPS39 expression constructs. Fluorescence microscopy (**E**) and quantification (**F**) of LDL-pHrodo Red (red) in A549 cells expressing mutant or WT ORF3a and empty vector. Scale bar = 100 µm. *N* = 3 biological replicates. One-way ANOVA. (**G**) Proposed model illustrating LDL acidification, and viral infection induced by Beta variant or wild-type ORF3a. We do not know where the PCSK9 interaction is taking place and if the binding interface with ORF3a is similar or different to that of VPS39 or LDLR and ORF3a. Data information: In (**B**, **F**), data are presented as mean ± SEM. ****P < 0.0001, ***P < 0.001, **P < 0.01, *P < 0.05. Source data are available online for this figure.

**Figure EV5:**
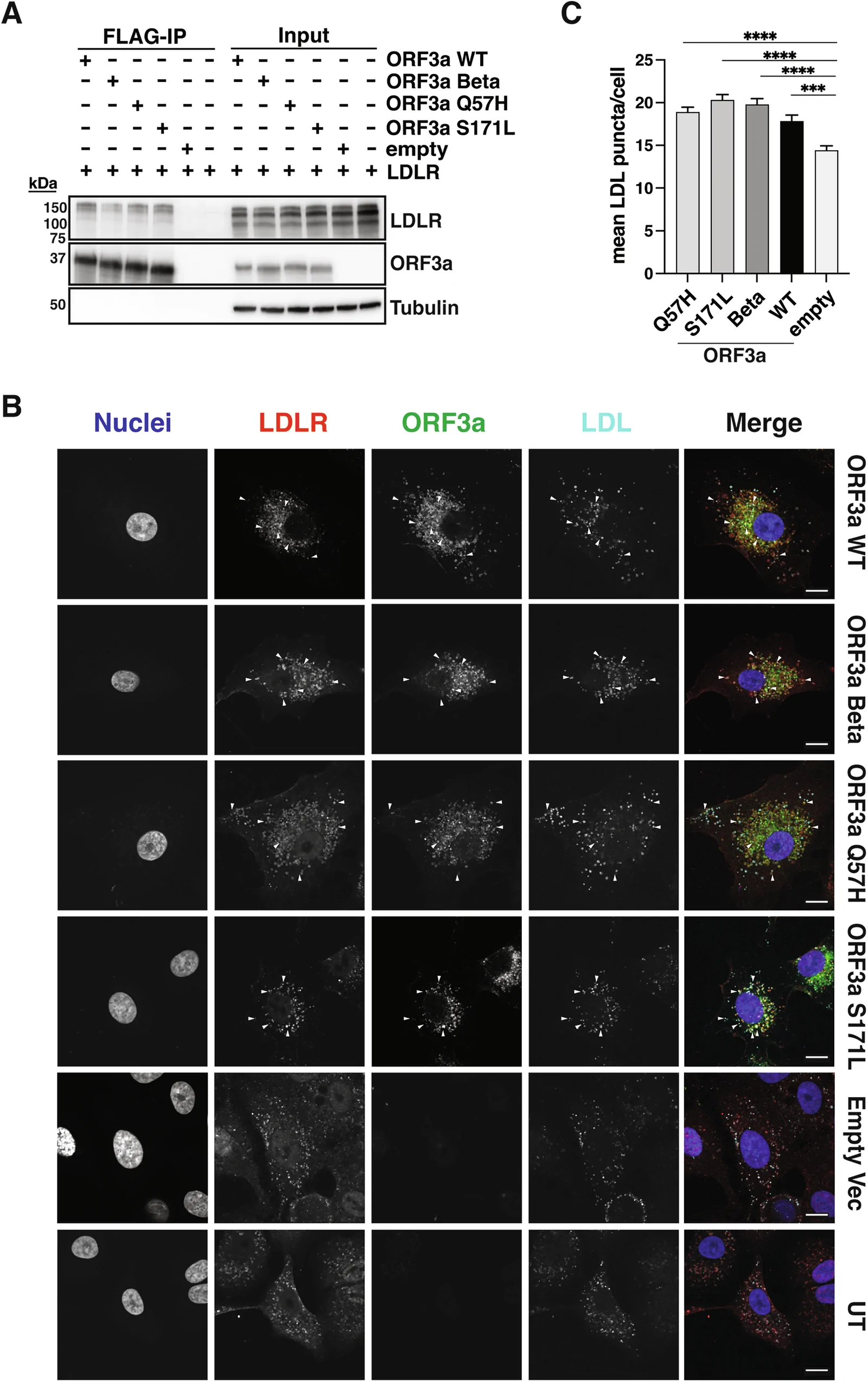
SARS-CoV-2 ORF3a interacts with host LDLR at endosomes. (**A**) Representative Western blot of anti-FLAG co-immunoprecipitation of HEK293T cells overexpressing GFP-LDLR and WT or mutant ORF3a-FLAG constructs. (**B**) Representative confocal microscopy images of A549 cells overexpressing WT or mutant ORF3a-FLAG constructs (green), stained for endogenous LDLR (red), and treated with fluorescently labeled LDL (cyan). Arrowheads indicate puncta containing LDLR, LDL, and ORF3a. Scale bar = 10 µm. (**C**) Quantification of LDL-pHrodo Red puncta in A549 cells expressing mutant or WT ORF3a, or empty vector. *N* = 3 biological replicates. One-way ANOVA. Data information: In (**C**), data are presented as mean ± SEM. ****P < 0.0001, ***P < 0.001.

**Figure EV6:**
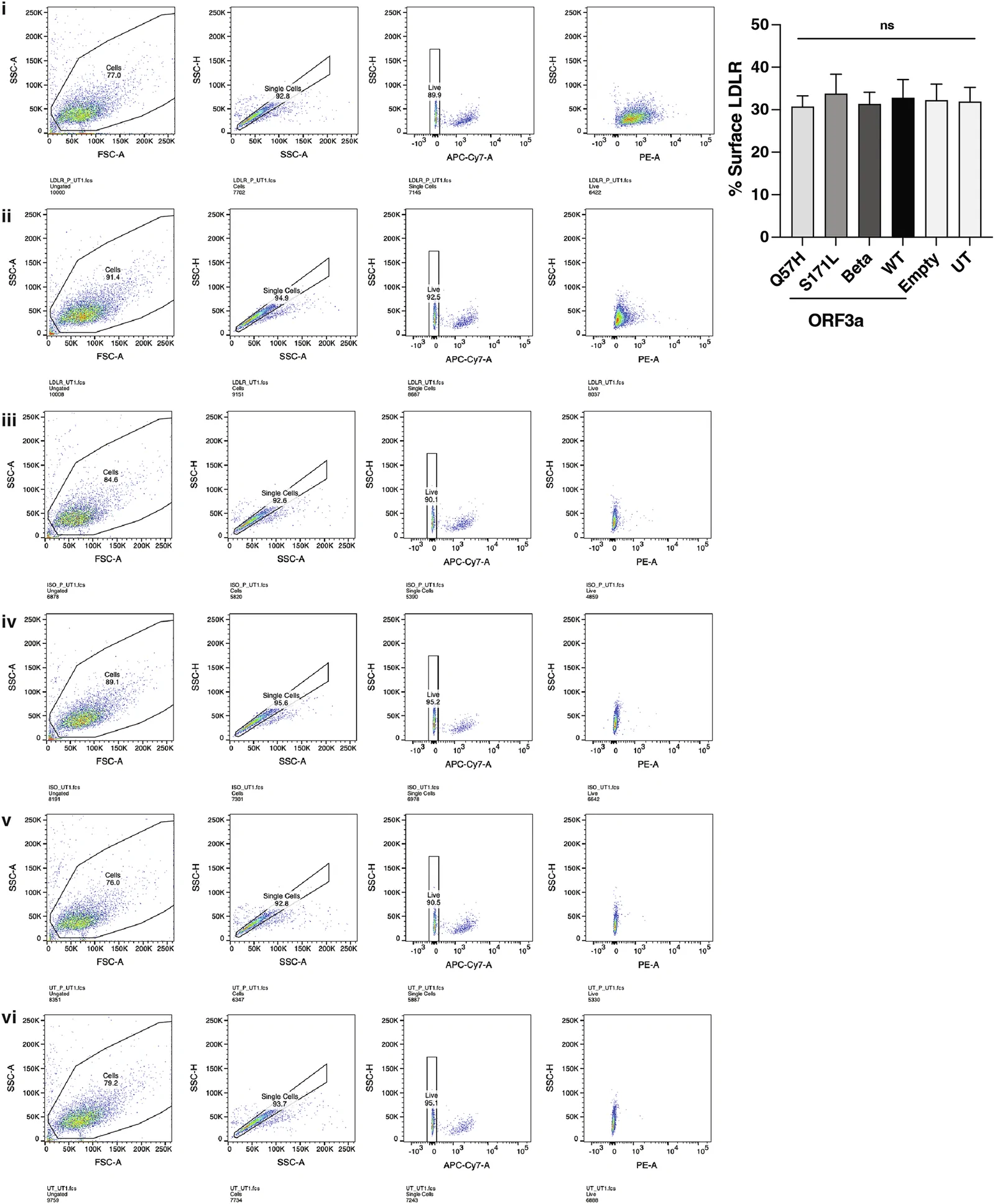
SARS-CoV-2 ORF3a does not significantly change LDLR surface presentation. Flow cytometry gating strategy to measure total versus surface LDLR protein abundance in no antibody (UT) control A549-ACE2 cells. Cells were stained with eFlour 780 viability dye, and anti-LDLR-PE (LDLR) or isotype control-PE (ISO) antibodies in the presence (P) or absence of 0.1% saponin permeabilization. Quantification of surface (no detergent) versus total (saponin) LDLR in A549-ACE2 cells expressing empty vector, WT or mutant ORF3a. *N* = 3 biological replicates. One-way ANOVA. Data information: In (bar graph), data are presented as mean ± SEM.

The Beta variant, which responded to LDL treatment with the largest increase in viral replication (Fig. 1H), contains two unique point mutations in ORF3a: Q57H and S171L (Fig. 4A). The WT- and Beta-ORF3a proteins effectively co-immunoprecipitated and colocalized with LDLR, as did the ORF3a proteins carrying either one of the two Beta point mutations (Fig. EV5A,B). In contrast, LDL uptake depended on the ORF3a genotype, with Beta and S171L ORF3a proteins enhancing cholesterol accumulation compared to control cells (Fig. EV5C). Similarly, we found that Calu-3 cell lines that stably expressed the WT or mutant ORF3a proteins enhanced infection by the ORF3a-STOP virus, with the Beta variant and the S171L ORF3a mutants showing the largest increases (Fig. 4B). Strikingly, the ORF3a genotype did not change the surface presentation of LDLR, supporting the model that receptor recycling through the CCC/WASH complexes remains unaltered (Fig. EV6).

We hypothesized that the enhanced LDL uptake and viral fitness conferred by Beta ORF3a was due to a secondary step downstream of LDLR engagement and recycling. To determine if Beta ORF3a bound host proteins differ from WT, we performed AP-MS using both proteins. This analysis identified VPS39–a member of the HOPS complex (Miao et al, 2021) and previously identified as a host interaction partner of ORF3a (Gordon et al, 2020b)–as highly enriched for binding to WT, but not Beta ORF3a (Fig. 4C). This was confirmed in co-immunoprecipitation experiments where binding of the FLAG-tagged Beta ORF3a to VPS39 was abrogated (Fig. 4D). Importantly, the S171L mutation alone weakened the interaction markedly, confirming that VPS39 binds to the C-terminus of ORF3a (Miller et al, 2023) (Fig. 4D).

As previously described, a function of the interaction of ancestral ORF3a and VPS39 is the deacidification of host endosomes (Chen et al, 2021)—an activity that likely diminishes cholesterol hydrolysis from LDL—we tested whether the point mutations in Beta ORF3a affect endosome acidity. A549 cells expressing the different ORF3a genotypes were treated with LDL-pHrodo red, an LDL particle with an intercalating dye that fluoresces in an acidic environment (Kemmer et al, 2015; Kitano et al, 2020; Zhang et al, 2022b). While LDL puncta were visible under all conditions, including in control cells transduced with the empty vector alone, WT ORF3a decreased the mean fluorescence intensity (MFI) of the puncta, consistent with its known deacidification activity (Fig. 4E,F). In contrast, the Beta and S171L mutants significantly increased the MFI of LDL-pHrodo Red, supporting the model that enhanced acidification of LDL-positive endosomes and more efficient LDL hydrolysis led to optimized LDL uptake by Beta variant mutants (Fig. 4E,F).

## Discussion

Our findings support a model in which the SARS-CoV-2 accessory protein ORF3a acts as an LDL uptake factor facilitating the availability of cholesterol for viral RNA replication. We further show that natural viral evolution in the Beta lineage has optimized the full usage of circulating cholesterol for enhancing viral infection (Fig. 4G). During infection with the WT virus, ORF3a binds and stabilizes LDLR at host endosomes by decreasing PCSK9 interactions, which leads to an increase in LDL uptake and enhanced genomic replication. However, due to endosomal deacidification by the WT ORF3a, the LDLR does not release its cholesterol optimally for cellular uses. In contrast, during Beta variant infection, the mutant ORF3a binds LDLR and restores endosome acidification, leading to optimized cholesterol uptake to better support viral infection. This provides a molecular link between physical conditions with high circulating cholesterol, such as hypercholesterolemia, obesity and severity of COVID-19, which was clinically striking in the early days of the pandemic (Bhaskaran et al, 2021; Bonetti et al, 2003; Collaboration NCDRF 2016; Cummings et al, 2020; Gao et al, 2021; Hamer et al, 2020; Klop et al, 2013; Tamara and Tahapary, 2020).

Previous studies have demonstrated that ORF3a localizes to lysosomes, damages their membrane stability, and prevents the acidification of endosomes (Bignon et al, 2022; Chen et al, 2021; Hayn et al, 2021; Kern et al, 2021; Miao et al, 2021; Michelucci et al, 2025; Solvik et al, 2022). Our data, combined with these findings suggest that ORF3a interacts with LDLR when ORF3a-containing, damaged lysosomes attempt to fuse with LDLR-containing incoming endosomes, but that the fusion remains ineffective for LDL hydrolysis. Alternatively, since SARS-CoV-2 has been proposed to cause lysosomal exocytosis for viral egress, the interaction of ORF3a with LDLR could occur at the plasma membrane before endocytosis (Chen et al, 2021; Ghosh et al, 2020). However, since we observe ORF3a and LDLR colocalizing intracellularly, we favor the hypothesis of an endosomal interaction.

Our data show that (1) SARS-CoV-2 infection increases with PCSK9 inhibition, (2) ORF3a enriches LDLR, (3) ORF3a decreases the PCSK9-LDLR interaction, and (4) ORF3a decreases endosomal acidification; however, the mechanisms by which ORF3a prevents the PCSK9-LDLR interaction remain unknown. We propose three complementary mechanisms. First, ORF3a-driven LDLR enrichment may crowd out PCSK9 binding in the endosomes; or ORF3a may sterically hinder the LDLR beta-propeller conformation, directly dislodging PCSK9 (Kwon et al, 2008; Lo Surdo et al, 2011). Second, since PCSK9 binding to LDLR blocks the conformational change required for SNX17-mediated recycling in acidic endosomes, ORF3a’s pH elevation may itself reduce PCSK9 binding (Guan et al, 2025). Third, ORF3a-induced dense body formation sequesters membranes and proteins from early endosomes and the *trans*-Golgi (Hartmann et al, 2025), two sites of known-PCSK9-LDLR interactions, likely disrupting structures that normally facilitate receptor degradation. Regardless of the precise mechanism, we propose that the ORF3a-LDLR interaction in the endosomes is primarily the site of PCSK9 inhibition.

SARS-CoV ORF3a is 73% identical to its SARS-CoV-2 counterpart, suggesting that it may also benefit from increased extracellular LDL (Zhang et al, 2022a). It also rearranges cellular membranes, interferes with intracellular trafficking, and is a proposed channel protein, suggesting that it could also enhance LDL uptake (Freundt et al, 2010; Lu et al, 2006; Yu et al, 2004). However, one major difference between the two proteins is an unstructured loop present in SARS-CoV-2 ORF3a that includes residue S171–a region that interacts with VPS39 and allows it to prevent lysosomal fusion or endosomal acidification (Miller et al, 2023). Future studies are needed to delineate the relevance of this newly defined viral-host interaction (ORF3a-LDLR) across different coronaviruses.

How the need for endosome acidification and deacidification is balanced during infection requires further studies. Notably, the ion channel functionality of ORF3a has been recently called into doubt (Miller et al, 2023). Instead, ORF3a has been suggested as a water-permeable channel that induces lysosomal swelling as a mechanism of deacidification (Michelucci et al, 2025). How S171L changes the water permissibility of ORF3a remains unknown and may explain its opposing deacidification profile in the Beta variant. Deacidification is thought to benefit viral egress (Chen et al, 2021; Ghosh et al, 2020) and that might explain why Q57H and S171L mutations were not maintained in other viral variants as they hinder this process. In addition, we speculate that as LDL uptake and hydrolysis may be linked to ORF3a’s role as a pathogenesis factor, the virus may not have evolved to strengthen this function as it is not beneficial for viral attenuation and survival of the host.

Our TEM experiments of infected cells showed potential morphological changes to endo/lysosomal structures (Fig. 2E,F). This is in line with previous reports which: (1) determined that the formation of ORF3a-induced dense bodies is dependent on the c-terminal domain containing S171L; however, how these structures relate to intracellular cholesterol requires further studies (Hartmann et al, 2025). (2) Another study identified ORF3a-Filipin III-LAMP1 positive structures and determined that NPC2 trafficking and subsequent liberation of lysosomal cholesterol was impaired by ORF3a (Goodson et al, 2026). We further show that different Orf3a genotypes affect endosomal/lysosomal acidification, which is a major function of that subcellular compartment (Fig. 4E,F). These observations strengthen our interpretation that the Beta variant has optimized LDL uptake and hydrolysis to improve its replication.

The decreased enhancement of LDL-mediated infection in the Omicron variant, which is less pathogenic but more transmissible, supports this model. The Omicron BA.1 variant has WT ORF3a and none of its subvariants carries the ORF3a mutations found in the Beta variant. However, a recent report determined that Omicron decreases ORF3a expression at the RNA and protein levels relative to the ancestral strain, which might explain the decrease in Omicron sensitivity to LDL (Bouhaddou et al, 2023). Notably, the T233I mutation in ORF3a in Omicron BA.2 and all its subvariants, including XBB.1.5 is an interesting candidate and for future studies.

Beyond ORF3a, other SARS-CoV-2 proteins (NSP1, NSP2, NSP6, NSP12) significantly decreased LDL uptake (Fig. 3C). Additionally, some viral proteins were not tested including NSP3, NSP9, NSP11, NSP16, and ORF7a, further exploration into their effects on LDL uptake is required. Yet during infection, LDL uptake is increased overall suggesting that the ORF3a effect is dominant. Notably, this effect may not exclusively be due to ORF3a which may work in concert with other viral proteins to enhance this benefit. Future studies should investigate how these viral proteins interact to collectively shape LDL uptake during infection.

## Methods

**Table:** Reagents and tools table

Reagent/resource	Reference or source	Identifier or catalog number
**Experimental models**		
A549 cells	Nevan Krogen	N/A
A549-ACE2 cells	Synthego	N/A
A549-ACE2 ∆ldlr	Synthego	N/A
A549-ACE2 ∆npc1	Synthego	N/A
A549-ACE2 ∆npc2	Synthego	N/A
HEK-293T cells	ATCC	CRL-3216
Calu3 cells	ATCC	HTB-55
BHK21 cells	ATCC	CCL-10
VERO-E6 cells	ATCC	CRL-1586
VERO-E6-ACE2/TMPRSS2 cells	Barney Graham	N/A
Golden Syrian hamster	Charles River	049LVG
SARS-CoV-2 WA1	BEI	NR-52281
SARS-CoV-2 Alpha	Charles Chiu/Raul Andino	N/A
SARS-CoV-2 Beta	BEI	NR-54008
SARS-CoV-2 Gamma	BEI	P.1
SARS-CoV-2 Delta	CA DPH	N/A
SARS-CoV-2 Lambda	BEI	NR-55654
SARS-CoV-2 Singapore	Siew Wai Fong, Laurent Renial, Lisa F.P. Ng	GSAID: EPI_ISL_414378
SARS-CoV-2 Omicron	Charles Chiu	N/A
**Recombinant DNA**		
PTV plasmids	Deng et al, 2021	N/A
VLP plasmids	Syed et al, 2021	Addgene: 177938, 17793, 177960, 177942
pGLUE Replicon plasmids	Taha et al, 2023	N/A
pST-SARS2-nsp1	This study	N/A
pST-SARS2-nsp2	This study	N/A
pST-SARS2-nsp4	This study	N/A
pST-SARS2-nsp5	This study	N/A
pST-SARS2-nsp6	This study	N/A
pST-SARS2-nsp7	This study	N/A
pST-SARS2-nsp8	This study	N/A
pST-SARS2-nsp9	This study	N/A
pST-SARS2-nsp10	This study	N/A
pST-SARS2-nsp12	This study	N/A
pST-SARS2-nsp13	This study	N/A
pST-SARS2-nsp14	This study	N/A
pST-SARS2-nsp15	This study	N/A
pST-SARS2-s	This study	N/A
pST-SARS2-orf3a	This study	N/A
pST-SARS2-orf3a Beta	This study	N/A
pST-SARS2-orf3a Q57H	This study	N/A
pST-SARS2-orf3a S171L	This study	N/A
pST-SARS2-orf3b	This study	N/A
pST-SARS2-e	This study	N/A
pST-SARS2-m	This study	N/A
pST-SARS2-orf6	This study	N/A
pST-SARS2-orf7b	This study	N/A
pST-SARS2-orf8	This study	N/A
pST-SARS2-orf9c	This study	N/A
pST-SARS2-n	This study	N/A
pST-SARS2-orf10	This study	N/A
pST-empty vector	This study	N/A
pST-vps39	This study	N/A
pST-vps35	This study	N/A
pST-ccdc22	This study	N/A
pST-snx17	This study	N/A
pBa-LSS-GFP-LDLR WT	Addgene	98184
**Antibodies**		
Mouse anti-dsRNA J2	Jena Biosciences	RNA-SCI-10010500
Mouse anti-FLAG M2	Sigma	F1804
Rabbit anti-LDLR	ProteinTech	10785-1-AP
Mouse anti-LDLR	ProteinTech	66414-1-1g
Rabbit anti-alpha-Tubulin	ProteinTech	80762-1-RR
Mouse anti-GAPDH	ProteinTech	16219-1-AP
Rabbit anti-VPS39	ProteinTech	16219-1-AP
Rabbit anti-PCSK9	ProteinTech	55206-1-AP
Rabbit anti-VPS35	ProteinTech	10236-1-AP
Rabbit anti-CCDC22	Abcam	Ab220438
Rabbit anti-SNX17	Cell Signaling Technology	75885
Mouse anti-LDLR-PE	R&D Systems	FAB2148P
Mouse IgG1 PE Isotype Control-PE	R&D Systems	IC002P
Goat anti-mouse-HRP	Bethyl	A90-516P
Goat anti-rabbit-HRP	Bethyl	A120-201P
**Oligonucleotides and other sequence-based reagents**		
SARS2-N5_F	AAATTTTGGGGACCAGGAAC	
SARS2-N5_R	AAATTTTGGGGACCAGGAAC	
SARS2-N5_Probe	FAM-ATGTCGCGCATTGGCATGGA-BHQ1	
SARS2-E_F	ACAGGTACGTTAATAGTTAATAGCGT	
SARS2-E_R	ATATTGCAGCAGTACGCACACA	
SARS2-E_Probe	FAM-ACACTAGCCATCCTTACTGCGCTTCG-BHQ1	
**Chemicals, enzymes and other reagents**		
FLAG M2-Magnetic Beads	Sigma	M8823
Dynabeads Protein A	Invitrogen	10001D
LDL	Invitrogen	L3486
DiI-LDL	Invitrogen	L3482
pHrodo-LDL	Invitrogen	L34356
HDL	Sigma	L8039
VLDL	Sigma	437647
Oleic Acid	Sigma	O1008
Cholesterol	Sigma	47127-U
(R)-IMPP	Caymen chemicals	20861
Hoechst	Invitrogen	62249
Cyclohexamide	Sigma-Aldrich	239763-M
LipidTox Deep Red	Invitrogen	H34477
Lipid Extraction Buffer	Abcam	ab211044
X-tremeGene9	Roche	XTG9-RO
Non-polar Lipid Mix 2 (Ctl B)	Caymen Chemicals	29377
**Software**		
GraphPad Prism 9.0	https://www.graphpad.com	
FlowJo v.10.6.2	https://www.flowjo.com	
Imaris 9.9.1	https://imaris.oxinst.com/	
MetaXpress 6.7.2.290	https://www.moleculardevices.com/products/cellular-imaging-systems/high-content-analysis/metaxpress	
ImageJ 1.54t	https://imagej.nih.gov/ij/index.html	
**Other**		

### Ethics statement

All animal experiments and procedures were conducted in accordance with the guidelines and policies of the Institutional Animal Care and Use Committee (IACUC) at the University of California, San Francisco (UCSF), under approved protocol #AN209208-00. All studies involving live SARS CoV-2 and infected Golden Syrian hamsters (*Mesocricetus auratus*) were performed in the Gladstone Animal Biosafety Level 3 (ABSL-3) facility under protocols approved by the UCSF Institutional Biosafety Committee (IBC) and authorized under Biosafety Use Authorization (BUA) #BU184109-05A.

### Cell lines

A549, HEK293T, HEK293T-ACE2/TMPRSS (Deng et al, 2021), BHK21, and Vero-ACE2/TMPRSS2 (Taha et al, 2023) cells were cultured in complete DMEM (DMEM with 10% Serum Plus II, 1% penicillin–streptomycin, 1% glutamine). A549-ACE2 cells were made as described (Wang et al, 2021a) and cultured in complete DMEM supplemented with 10 µg/mL blasticidin. Calu-3 cells were cultured in Advanced MEM (MEM with 10% fetal bovine serum, 1% penicillin–streptomycin, 1% GlutaMax). Cell lines were routinely tested for mycoplasma contamination.

### Culture of human airway organoids (HAOs)

To generate HAOs, input cells were used from whole-lung lavages or from digestion of whole lungs. HAOs were generated from these cells as previously described (Sachs et al, 2019; Zhou et al, 2018). Briefly, single cells were suspended in 65% reduced growth factor BME2 (Basement Membrane Extract, Type 2, Trevigen, catalogue number 3533-001-02). From this mixture, 50 µL drops containing 1000–40,000 cells were seeded in 24-well suspension culture plates (GreinerBio-one, catalogue number 662-102). Drops were incubated at 37 °C for >20 min and solidified. After this, 500 µL of HAO media was added to each well. HAO media is organoid basal media (Advanced DMEM/F12 supplemented with 1% penicillin–streptomycin, 1% Glutamax, and 10 mM HEPES) supplemented with 10% (vol/vol) R-spondin1 conditioned media, 1% B27 (Gibco), 25 ng/mL Noggin (Peprotech), 1.25 mM N-Acetylcysteine (Sigma-Aldrich), 10 mM Nicotinamide (Sigma-Aldrich), 5 nM Herefulin beta-1 (Peprotech), and 100 µg/mL Primocin (InvivoGen). HAO media was further supplemented with 5 µM Y-27632, 500 nM A83-01, 500 nM SB202190, 25 ng/mL FGF-7, 100 ng/mL FGF-10 (all from Stem Cell Technologies), and HAO media was replaced every 3–4 days.

After 14–21 days, organoids were passaged. For this, cold basal media was used to collect organoids in 15 ml Falcon tubes and dissolve BME2. Tubes were centrifuged at 250×*g* for 5 min at 4 °C. Media was aspirated, 10x TrypLE Select (Gibco) was added to the organoids, and the mixture was incubated at 37 °C for 5–10 min. Organoids were further dissociated by pipette mixing and then diluted in cold basal media. After another spin and media aspiration, cells were mixed with BME2 and seeded into new drops. After this initial passage, organoids were passaged every 10–21 days. Stocks of early-passage (P1-P3) organoid lines were prepared by dissociating organoids, mixing them with recovery cell culture freezing media (Gibco), and freezing them by standard procedures. These samples could be thawed and immediately cultured in HAO media.

### Viral infection

In total, 30,000 A549-ACE2 cells (acquired from Synthego) were plated into black-wall 96-well plates. For infection, DMEM with 5% serum plus for infection was used with or without 4 µg/ml LDL (ThermoFisher), 4 µg/ml HDL (Sigma), or 10 µM oleic acid (Sigma). Infections were carried out at an MOI of 0.1 as measured by plaque assays, for 72 h at 37 °C. Cells were fixed with 4% PFA for ≥30 min at RT or in TRIZOL-RNA STAT60.

### Chemical induction of LDLR

In total, 30,000 A549-ACE2 cells (acquired from Synthego) were plated into black wall-96-well plates. Twenty-four hours prior to infection, cells were treated with 0, 0.78, 6.25, and 25 µM (R)-IMPP (Cayman Chemicals) and 4 µg/ml DiI-LDL (ThermoFisher). Infections were carried out at an MOI of 0.1 as measured by plaque assays, for 72 h at 37 °C. Cells were fixed with 4% PFA for ≥30 min at RT.

### Quantification of viral transcripts

RNA was extracted using an RNA extraction kit (Zymo), cDNA synthesized, and 1 ng of cDNA used for quantitative PCR (qPCR) with previously published Taqman Primers, Probes, and N or E standard curves (Fozouni et al, 2021).

### Detection of viral infection

Cells were permeabilized with 0.1% TritonX-100, blocked with 3% BSA in PBS, and stained with primary antibodies (J2 Ms-anti-dsRNA, 1:200), secondary antibodies (Goat-anti-Ms-AF647, 1:500), and Hoechst nuclear stain (1:2000). Cells were imaged and analyzed by high content imaging, Molecular Devices ImageXpress Confocal microscope at ×10 or ×40. The fluorescence of dsRNA puncta was used to identify infected cells.

### Plasmid generation

Plasmids were cloned by PCR amplification of genes from previously published plasmids encoding the SARS-CoV-2 orfeome (Gordon et al, 2020b). Vector backbone is a modified pLVX302 plasmid expressing BFP, using an EF1a promoter to express the FLAG tagged viral protein. Viral genes were inserted into the vector backbone by HiFi DNA Assembly (NEB). Site directed mutagenesis (NEB) was used to make point mutants in ORF3a. These plasmids were combined with packing and envelope expressing plasmids and transfected into HEK293Ts with XtremeGene9 (Sigma-Aldrich). Supernatant was collected and quantified using LentiX-p24 titration assays (Takara Biosciences). A549 cells were transduced for 48 h with subsequent drug selection for stable expressing cell lines.

### Replicon assay

In all, 100,000 BHK21 cells were plated in 12-well plates, transfected with 2 µg of plasmids with a 3:1 ratio of XtremeGene9 (Sigma) incubated at RT for 30–60 min. Twenty-four hours post transfection, media was washed and replaced with fresh media. Seventy-two hours post transfection, supernatant was collected and filtered through a 0.45 µm PVDF membrane. In all, 10,000 A549-ACE2 or Vero-ACE2/TMPRSS2 cells in a 96-well plate were infected with filtered supernatant, nanoluciferase was measured 48 h post infection.

### Correlative light and electron microscopy

In total, 500,000 Vero-ACE2/TMPRSS cells were grown for correlative light and electron microscopy on gridded 35 mm MatTek glass-bottom dishes (#P35G-1.5-14-C-GRD MatTek Corp., Ashland, MA, USA). Cells were infected with 10 µl single-round mNeonGreen in the presence or absence of LDL for 24 h. Cells were fixed in 4% PFA in PBS for 10 min at RT and stained with Hoechst for nuclei. Following fluorescence imaging, cells were fixed in 2.5% glutaraldehyde and 2.5% paraformaldehyde in PBS, pH 7.2. Samples were rinsed (3×; 5 min, Room Temperature) in 1× PBS and immersed in 1% osmium tetroxide with 1.6% potassium ferricyanide in 1× PBS for 30 min. Samples were rinsed (3×; 5 min, RT) in buffer and briefly washed with distilled water (1×; 1 min, RT) before sample were then subjected to an ascending ethanol gradient followed by pure ethanol. Samples were progressively infiltrated (using ethanol as the solvent) with Epon resin (EMS, Hatfield, PA, USA) and polymerized at 60 °C for 24–48 h. Care was taken to ensure only a thin amount of resin remained within the glass bottom dishes to enable the best possible chance for separation of the glass coverslip. Following polymerization, the glass coverslips were removed using ultra-thin Personna razor blades (EMS, Hatfield, PA, USA) and liquid nitrogen exposure, as needed. To enable correlative light and electron microscopy, specific cells, identified by the gridded alpha-numerical labeling on the plates documented during fluorescence imaging, were carefully removed, precisely trimmed and mounted on a blank resin block with cyanoacrylate glue for sectioning. Serial thin sections (80 nm) were cut using a Leica UC6 ultramicrotome (Leica, Wetzlar, Germany) from the surface of the block until ~4–5 microns in to ensure complete capture of the cell volumes. Section-ribbons were then collected sequentially onto formvar-coated slot grids. The grids were post-stained with 2% uranyl acetate followed by Reynold’s lead citrate, for 5 min each. The sections were imaged using a FEI Tecnai 12 120 kV TEM (FEI, Hillsboro, OR, USA) and data recorded using a Gatan Rio 16 CMOS camera with Gatan Microscopy Suite software (Gatan Inc., Pleasanton, CA, USA).

### Co-immunoprecipitation

HEK293T cells were transfected with respective plasmids and XtremeGene9 (1:3; Sigma) for 72 h. Cells were collected, protein quantified by DC Assay (Bio-Rad), and 2 µg of protein was loaded onto 100 µl of M2 FLAG magnetic beads (Sigma) or Protein A Dynabeads (Invitrogen; incubated with Rb-anti-LDLR (ProteinTech) for 15 min rotating at RT) for 18–24 h at 4 °C while rotating. Beads were washed six times, eluted with 3× FLAG peptide (Sigma) for 30 min at 4 °C shaking or incubated with loading buffer for 10 min at 70 °C shaking, 5 µg of input and IP were loaded onto a Criterion acrylamide gel (Bio-Rad) for Western blot analysis.

### Localization of ORF3a and LDLR by immunofluorescence

HEK293T cells were transfected with ORF3a-FLAG and pBa-LSS-GFP-LDLR WT (Addgene #98184) expressing plasmids as above for 48 h. Cells were lifted and plated onto poly-L-lysine treated coverslips for 24 h. Wells were treated with DiI-LDL as described below, fixed with 4% paraformaldehyde for 10 min, permeabilized with 0.1% TritonX-100 for 10 min, and blocked with 3% BSA in PBS for 30 min. Coverslips were stained with primary antibodies (Ms-anti-M2 FLAG, 1:200), secondary antibodies (Goat-anti-Ms-AF647, 1:500), and Hoechst nuclear stain (1:500). Coverslips were imaged at ×60 using an Olympus FV3000RS scanning confocal microscope. Imaris 9.9.1 Colocalization function was used for quantification.

### Low density lipoprotein uptake assay

Infected A549-ACE2 cells were treated with 4 µg/ml DiI-LDL (ThermoFisher) at 68 hpi for 4 h at 37 °C. Transduced A549 cells were treated with 4 µg/ml DiI-LDL (ThermoFisher) for 4 h at 37 °C. Treated cells were washed with PBS and fixed with 4% paraformaldehyde in PBS for 10 min, cells were washed repeatedly with PBS, counterstained with Hoechst (1:500) and dsRNA (infected cells, as above), imaged and analyzed by high content imaging on a Molecular Devices ImageXpress Confocal microscope at ×10 and ×40. DiI-LDL puncta were counted by the granularity analysis and relative number of puncta to uninfected/untransduced A549 cells was calculated.

### Low density lipoprotein acidification assay

Transduced A549 cells were treated with 4 µg/ml LDL-pHrodo Red (ThermoFisher) for 4 h at 37 °C. Treated cells were washed repeatedly with HBSS, counterstained with Hoechst dye (ThermoFisher), and live imaged and analyzed by high content imaging, Molecular Devices ImageXpress Confocal microscope at ×10. LDL-pHrodo Red puncta were counted and scored by the granularity analysis. Relative number of puncta to untransduced A549 cells was calculated.

### Total cellular cholesterol assay

Cells were fixed with fixation buffer (4% paraformaldehyde and 1% methanol in Tris-HCl Buffer) for 10 min, washed with Tris-HCl Buffer with 1% Tween20, and detected in Filipin III in Tris-HCl (1:100, Cayman Chemicals) for 1 h. Samples were washed as above, imaged and analyzed by high content imaging as described above. Fluorescent intensity of Filipin III staining was measured and compared to that of untransduced A549 cells was calculated.

### Thin-layer chromatography

Equal number of cells were collected, lipids extracted with Extraction Buffer (hexanes-isopropanol; 3:2; Abcam) for 30 min with vortexing, samples spun down, solubilized lipids collected in supernatant, dried down at 37 °C. Samples were resuspended in chloroform-methanol (2:1), HPTLC silicagel60 plate (Supelco) primed with dichloromethane: methanol (1:1), samples loaded, run with a Mangold mobile phase (petroleum ether-diethyl ether-glacial acetic acid; 40:10:0.5), developed in 5% phosphomolybdic acid in ethanol with heating at 120 °C for 30 min, densitometry performed in ImageJ 1.51 s. Lipid standards diluted in chloroform-methanol (2:1) to 40 µg/µl, 2.5 µl spotted per lane. Control A (Cholesterol solution; Sigma) and Control B (Non-polar Lipid Mixture 2; Cayman Chemicals).

### Protein stability assays

HEK293T cells were co-transfected with GFP-LDLR and wild-type ORF3a or empty vector for 48 h. Samples were treated with either DMSO or 50 µg/ml cycloheximide for 3, 6, 12, or 24 h. Protein samples were collected, quantified by DC assay (Bio-Rad), analyzed by Western blot, and band intensities were measured by ImageJ.

### Golden Syrian hamster infections

Eight-week-old male hamsters (Charles River) were fed high cholesterol (15% lard, 0.5% cholesterol) or normal chow for 2 weeks. Animals were intranasally infected with 10^3^ PFU/animal of SARS-CoV-2 WA1 or ORF3a-STOP for 48 h. Tissues were collected for lipid analysis by TLC, plaque assays, and histology (HistoWiz). Immunohistochemistry (HistoWiz) with anti-Nucleocapsid antibodies (GeneTex), area and intensity of IHC were measured using Fiji 17-13-18-405. *N* = 5 animals per group. All animals were housed at AAALAC accredited ABSL3 facility of Gladstone Institutes. The experiment was carried out in accordance with the UCSF Institutional Animal Care and Use Committee (IACUC) and the recommendations of the Panel on Euthanasia of the American Veterinary Medical Association. All protocols concerning animal use were approved (AN209208-00) by the Institutional Animal Care and Use committees at the University of California, San Francisco and Gladstone Institutes and conducted in strict accordance with the National Institutes of Health Guide for the Care and Use of Laboratory Animal (Council, 2011). Golden Syrian hamsters were housed in a temperature- and humidity-controlled pathogen-free facility with 12-h light/dark cycle and ad libitum access to water and standard laboratory rodent chow.

### LDLR surface expression

Transduced A549-ACE2 cells were transduced with lentiviruses with wild-type or mutant ORF3a or empty vector controls. Cells were lifted with TryplE (Gibco), stained with eFluor780 viability dye (Invitrogen eBiosciences), and anti-LDLR-PE (LDLR) or isotype control-PE (ISO) antibodies (R&D Systems) in the presence (P) or absence of 0.1% saponin permeabilization. Surface and total LDLR staining was measured on a BD Fortessa X-20 and analyzed with FlowJo.

### Affinity purified mass spectrometry

HEK293T cells were transfected with wild-type or Beta variant ORF3a or empty vector for 48 h. Samples were collected, anti-FLAG purification, and analyzed by mass spectrometry as previously described (Gordon et al, 2020b).

### Quantification and statistical analysis

The number of experiments and replicates are indicated in individual figure legends. Data were processed and visualized using GraphPad Prism. All quantified data are represented mean ± SEM, as indicated, and quantification details are available in figure legends. Western blot band intensities were measured by ImageJ. For animal experiments, to estimate the minimum number of animals needed to reliably get the desired statistical significance (*P* < 0.05) we performed power analysis and determined the animal number required for the experiment.

## Supplementary information

Peer Review File

Source data Fig. 1

Source data Fig. 2

Source data Fig. 3

Source data Fig. 4

Expanded View Figures

## Data Availability

No large datasets relevant to the study have been deposited. The source data of this paper are collected in the following database record: biostudies:S-SCDT-10_1038-S44319-026-00907-0.

## References

[CR1] Abrams ME, Johnson KA, Perelman SS, Zhang LS, Endapally S, Mar KB, Thompson BM, McDonald JG, Schoggins JW, Radhakrishnan A et al (2020) Oxysterols provide innate immunity to bacterial infection by mobilizing cell surface accessible cholesterol. Nat Microbiol 5:929–942 10.1038/s41564-020-0701-5 PMC7442315 32284563

[CR2] Abu-Raddad LJ, Chemaitelly H, Ayoub HH, Yassine HM, Benslimane FM, Al Khatib HA, Tang P, Hasan MR, Coyle P, AlMukdad S et al (2022) Severity, criticality, and fatality of the severe acute respiratory syndrome coronavirus 2 (SARS-CoV-2) beta variant. Clin Infect Dis 75:e1188–e1191 10.1093/cid/ciab909 PMC9402694 34657152

[CR3] Arshad N, Laurent-Rolle M, Ahmed WS, Hsu JC, Mitchell SM, Pawlak J, Sengupta D, Biswas KH, Cresswell P (2023) SARS-CoV-2 accessory proteins ORF7a and ORF3a use distinct mechanisms to down-regulate MHC-I surface expression. Proc Natl Acad Sci USA 120:e2208525120 10.1073/pnas.2208525120 PMC9910621 36574644

[CR4] Bartuzi P, Billadeau DD, Favier R, Rong S, Dekker D, Fedoseienko A, Fieten H, Wijers M, Levels JH, Huijkman N et al (2016) CCC- and WASH-mediated endosomal sorting of LDLR is required for normal clearance of circulating LDL. Nat Commun 7:10961 10.1038/ncomms10961 PMC4792963 26965651

[CR5] Bhaskaran K, Bacon S, Evans SJ, Bates CJ, Rentsch CT, MacKenna B, Tomlinson L, Walker AJ, Schultze A, Morton CE et al (2021) Factors associated with deaths due to COVID-19 versus other causes: population-based cohort analysis of UK primary care data and linked national death registrations within the OpenSAFELY platform. Lancet Reg Health Eur 6:100109 10.1016/j.lanepe.2021.100109 PMC8106239 33997835

[CR6] Bignon E, Marazzi M, Monari A (2022) Hijacking of cellular functions by severe acute respiratory syndrome coronavirus-2. Permeabilization and polarization of the host lipid membrane by viroporins. J Phys Chem Lett 13:4642–4649 10.1021/acs.jpclett.2c01102 35593652

[CR7] Bonetti PO, Lerman LO, Lerman A (2003) Endothelial dysfunction: a marker of atherosclerotic risk. Arterioscler Thromb Vasc Biol 23:168–175 10.1161/01.atv.0000051384.43104.fc 12588755

[CR8] Bouhaddou M, Reuschl AK, Polacco BJ, Thorne LG, Ummadi MR, Ye C, Rosales R, Pelin A, Batra J, Jang GM et al (2023) SARS-CoV-2 variants evolve convergent strategies to remodel the host response. Cell 186:4597–4614.e4526 10.1016/j.cell.2023.08.026 PMC10604369 37738970

[CR9] Brown MS, Goldstein JL (2009) Cholesterol feedback: from Schoenheimer’s bottle to Scap’s MELADL. J Lipid Res 50(Suppl):S15–S27 10.1194/jlr.R800054-JLR200 PMC2674699 18974038

[CR10] Chen D, Zheng Q, Sun L, Ji M, Li Y, Deng H, Zhang H (2021) ORF3a of SARS-CoV-2 promotes lysosomal exocytosis-mediated viral egress. Dev Cell 56:3250–3263.e3255 10.1016/j.devcel.2021.10.006 PMC8502680 34706264

[CR11] Cohen C, Kleynhans J, von Gottberg A, McMorrow ML, Wolter N, Bhiman JN, Moyes J, du Plessis M, Carrim M, Buys A et al (2022) SARS-CoV-2 incidence, transmission, and reinfection in a rural and an urban setting: results of the PHIRST-C cohort study, South Africa, 2020-21. Lancet Infect Dis 22:821–834 10.1016/S1473-3099(22)00069-X PMC8920516 35298900

[CR12] Collaboration NCDRF (2016) Trends in adult body-mass index in 200 countries from 1975 to 2014: a pooled analysis of 1698 population-based measurement studies with 19.2 million participants. Lancet 387:1377–1396. 10.1016/S0140-6736(16)30054-X PMC7615134 27115820

[CR13] Cummings MJ, Baldwin MR, Abrams D, Jacobson SD, Meyer BJ, Balough EM, Aaron JG, Claassen J, Rabbani LE, Hastie J et al (2020) Epidemiology, clinical course, and outcomes of critically ill adults with COVID-19 in New York City: a prospective cohort study. Lancet 395:1763–1770 10.1016/S0140-6736(20)31189-2 PMC7237188 32442528

[CR14] Cummings RD, Kornfeld S, Schneider WJ, Hobgood KK, Tolleshaug H, Brown MS, Goldstein JL (1983) Biosynthesis of N- and O-linked oligosaccharides of the low density lipoprotein receptor. J Biol Chem 258:15261–15273 6317691

[CR15] Daniels LB, Sitapati AM, Zhang J, Zou J, Bui QM, Ren J, Longhurst CA, Criqui MH, Messer K (2020) Relation of statin use prior to admission to severity and recovery among COVID-19 inpatients. Am J Cardiol 136:149–155 10.1016/j.amjcard.2020.09.012 PMC7492151 32946859

[CR16] Daniloski Z, Jordan TX, Wessels HH, Hoagland DA, Kasela S, Legut M, Maniatis S, Mimitou EP, Lu L, Geller E et al (2021) Identification of required host factors for SARS-CoV-2 infection in human cells. Cell 184:92–105.e116 10.1016/j.cell.2020.10.030 PMC7584921 33147445

[CR17] Davies SP, Mycroft-West CJ, Pagani I, Hill HJ, Chen YH, Karlsson R, Bagdonaite I, Guimond SE, Stamataki Z, De Lima MA et al (2021) The hyperlipidaemic drug fenofibrate significantly reduces infection by SARS-CoV-2 in cell culture models. Front Pharm 12:660490 10.3389/fphar.2021.660490 PMC8377159 34421587

[CR18] Davis CG, Elhammer A, Russell DW, Schneider WJ, Kornfeld S, Brown MS, Goldstein JL (1986) Deletion of clustered O-linked carbohydrates does not impair function of low density lipoprotein receptor in transfected fibroblasts. J Biol Chem 261:2828–2838 3005267

[CR19] Davis CG, Goldstein JL, Sudhof TC, Anderson RG, Russell DW, Brown MS (1987) Acid-dependent ligand dissociation and recycling of LDL receptor mediated by growth factor homology region. Nature 326:760–765 10.1038/326760a0 3494949

[CR20] de Wilde AH, Jochmans D, Posthuma CC, Zevenhoven-Dobbe JC, van Nieuwkoop S, Bestebroer TM, van den Hoogen BG, Neyts J, Snijder EJ (2014) Screening of an FDA-approved compound library identifies four small-molecule inhibitors of Middle East respiratory syndrome coronavirus replication in cell culture. Antimicrob Agents Chemother 58:4875–4884 10.1128/AAC.03011-14 PMC4136071 24841269

[CR21] Deng X, Garcia-Knight MA, Khalid MM, Servellita V, Wang C, Morris MK, Sotomayor-Gonzalez A, Glasner DR, Reyes KR, Gliwa AS et al (2021) Transmission, infectivity, and neutralization of a spike L452R SARS-CoV-2 variant. Cell 184:3426–3437.e3428 10.1016/j.cell.2021.04.025 PMC8057738 33991487

[CR22] Ehrlich A, Ioannidis K, Nasar M, Abu Alkian I, Daskal Y, Atari N, Kliker L, Rainy N, Hofree M, Shafran Tikva S et al (2023) Efficacy and safety of metabolic interventions for the treatment of severe COVID-19: in vitro, observational, and non-randomized open-label interventional study. eLife 12:e79946 10.7554/eLife.79946 PMC9937660 36705566

[CR23] Fessler MB, Summer RS (2016) Surfactant lipids at the host-environment interface. Metabolic sensors, suppressors, and effectors of inflammatory lung disease. Am J Respir Cell Mol Biol 54:624–635 10.1165/rcmb.2016-0011PS PMC4942198 26859434

[CR24] Fozouni P, Son S, Diaz de Leon Derby M, Knott GJ, Gray CN, D’Ambrosio MV, Zhao C, Switz NA, Kumar GR, Stephens SI et al (2021) Amplification-free detection of SARS-CoV-2 with CRISPR-Cas13a and mobile phone microscopy. Cell 184:323–333.e329 10.1016/j.cell.2020.12.001 PMC7834310 33306959

[CR25] Freundt EC, Yu L, Goldsmith CS, Welsh S, Cheng A, Yount B, Liu W, Frieman MB, Buchholz UJ, Screaton GR et al (2010) The open reading frame 3a protein of severe acute respiratory syndrome-associated coronavirus promotes membrane rearrangement and cell death. J Virol 84:1097–1109 10.1128/JVI.01662-09 PMC2798367 19889773

[CR26] Gao M, Piernas C, Astbury NM, Hippisley-Cox J, O’Rahilly S, Aveyard P, Jebb SA (2021) Associations between body-mass index and COVID-19 severity in 6.9 million people in England: a prospective, community-based, cohort study. Lancet Diab Endocrinol 9:350–359 10.1016/S2213-8587(21)00089-9 PMC8081400 33932335

[CR27] Ghosh S, Dellibovi-Ragheb TA, Kerviel A, Pak E, Qiu Q, Fisher M, Takvorian PM, Bleck C, Hsu VW, Fehr AR et al (2020) Beta-coronaviruses use lysosomes for egress instead of the biosynthetic secretory pathway. Cell 183:1520–1535.e1514 10.1016/j.cell.2020.10.039 PMC7590812 33157038

[CR28] Goodson BA, Montenegro Vazquez V, Doyle A, Kolaczkowski OM, Liu R, Jia J, Ishida M, Ye C, Kell AM, Bradfute SB et al (2026) SARS-CoV-2 ORF3a blocks lysosomal cholesterol egress by disrupting VPS39-regulated NPC2 trafficking and BMP metabolism. Cell Rep 45:117544 10.1016/j.celrep.2026.117544 PMC13378056 42287635

[CR29] Gordon DE, Hiatt J, Bouhaddou M, Rezelj VV, Ulferts S, Braberg H, Jureka AS, Obernier K, Guo JZ, Batra J et al (2020a) Comparative host-coronavirus protein interaction networks reveal pan-viral disease mechanisms. Science 370:eabe9403 10.1126/science.abe9403 PMC7808408 33060197

[CR30] Gordon DE, Jang GM, Bouhaddou M, Xu J, Obernier K, White KM, O’Meara MJ, Rezelj VV, Guo JZ, Swaney DL et al (2020b) A SARS-CoV-2 protein interaction map reveals targets for drug repurposing. Nature 583:459–468 10.1038/s41586-020-2286-9 PMC7431030 32353859

[CR31] Gotto AM Jr (1990) Interrelationship of triglycerides with lipoproteins and high-density lipoproteins. Am J Cardiol 66:20A–23A 10.1016/0002-9149(90)90565-i 2203246

[CR32] Gowdy KM, Fessler MB (2013) Emerging roles for cholesterol and lipoproteins in lung disease. Pulm Pharm Ther 26:430–437 10.1016/j.pupt.2012.06.002 PMC3466369 22706330

[CR33] Grundy SM (1983) Absorption and metabolism of dietary cholesterol. Annu Rev Nutr 3:71–96 10.1146/annurev.nu.03.070183.000443 6357243

[CR34] Guan Y, Liu X, Yang Z, Zhu X, Liu M, Du M, Pan X, Wang Y (2025) PCSK9 promotes LDLR degradation by preventing SNX17-mediated LDLR recycling. Circulation 151:1512–1526 10.1161/CIRCULATIONAHA.124.072336 40071387

[CR35] Hamer M, Gale CR, Kivimaki M, Batty GD (2020) Overweight, obesity, and risk of hospitalization for COVID-19: a community-based cohort study of adults in the United Kingdom. Proc Natl Acad Sci USA 117:21011–21013 10.1073/pnas.2011086117 PMC7474583 32788355

[CR36] Hartmann S, Radochonski L, Ye C, Martinez-Sobrido L, Chen J (2025) SARS-CoV-2 ORF3a drives dynamic dense body formation for optimal viral infectivity. Nat Commun 16:4393 10.1038/s41467-025-59475-x PMC12069715 40355429

[CR37] Hayn M, Hirschenberger M, Koepke L, Nchioua R, Straub JH, Klute S, Hunszinger V, Zech F, Prelli Bozzo C, Aftab W et al (2021) Systematic functional analysis of SARS-CoV-2 proteins uncovers viral innate immune antagonists and remaining vulnerabilities. Cell Rep 35:109126 10.1016/j.celrep.2021.109126 PMC8078906 33974846

[CR38] Hoffmann HH, Sanchez-Rivera FJ, Schneider WM, Luna JM, Soto-Feliciano YM, Ashbrook AW, Le Pen J, Leal AA, Ricardo-Lax I, Michailidis E et al (2021) Functional interrogation of a SARS-CoV-2 host protein interactome identifies unique and shared coronavirus host factors. Cell Host Microbe 29:267–280.e265 10.1016/j.chom.2020.12.009 PMC7833927 33357464

[CR39] Ikonen E (2008) Cellular cholesterol trafficking and compartmentalization. Nat Rev Mol Cell Biol 9:125–138 10.1038/nrm2336 18216769

[CR40] Ishibashi S, Brown MS, Goldstein JL, Gerard RD, Hammer RE, Herz J (1993) Hypercholesterolemia in low density lipoprotein receptor knockout mice and its reversal by adenovirus-mediated gene delivery. J Clin Invest 92:883–893 10.1172/JCI116663 PMC294927 8349823

[CR41] Kemmer GC, Bogh SA, Urban M, Palmgren MG, Vosch T, Schiller J, Gunther Pomorski T (2015) Lipid-conjugated fluorescent pH sensors for monitoring pH changes in reconstituted membrane systems. Analyst 140:6313–6320 10.1039/c5an01180a 26280031

[CR42] Kern DM, Sorum B, Mali SS, Hoel CM, Sridharan S, Remis JP, Toso DB, Kotecha A, Bautista DM, Brohawn SG (2021) Cryo-EM structure of SARS-CoV-2 ORF3a in lipid nanodiscs. Nat Struct Mol Biol 28:573–582 10.1038/s41594-021-00619-0 PMC8772433 34158638

[CR43] Kitano VJF, Ohyama Y, Hayashida C, Ito J, Okayasu M, Sato T, Ogasawara T, Tsujita M, Kakino A, Shimada J et al (2020) LDL uptake-dependent phosphatidylethanolamine translocation to the cell surface promotes fusion of osteoclast-like cells. J Cell Sci 133:jcs243840 10.1242/jcs.243840 32295848

[CR44] Klop B, Elte JW, Cabezas MC (2013) Dyslipidemia in obesity: mechanisms and potential targets. Nutrients 5:1218–1240 10.3390/nu5041218 PMC3705344 23584084

[CR45] Knoops K, Kikkert M, Worm SH, Zevenhoven-Dobbe JC, van der Meer Y, Koster AJ, Mommaas AM, Snijder EJ (2008) SARS-coronavirus replication is supported by a reticulovesicular network of modified endoplasmic reticulum. PLoS Biol 6:e226 10.1371/journal.pbio.0060226 PMC2535663 18798692

[CR46] Kulseth MA, Berge KE, Bogsrud MP, Leren TP (2010) Analysis of LDLR mRNA in patients with familial hypercholesterolemia revealed a novel mutation in intron 14, which activates a cryptic splice site. J Hum Genet 55:676–680 10.1038/jhg.2010.87 20703241

[CR47] Kwon HJ, Lagace TA, McNutt MC, Horton JD, Deisenhofer J (2008) Molecular basis for LDL receptor recognition by PCSK9. Proc Natl Acad Sci USA 105:1820–1825 10.1073/pnas.0712064105 PMC2538846 18250299

[CR48] Li GM, Li YG, Yamate M, Li SM, Ikuta K (2007) Lipid rafts play an important role in the early stage of severe acute respiratory syndrome-coronavirus life cycle. Microbes Infect 9:96–102 10.1016/j.micinf.2006.10.015 PMC7110773 17194611

[CR49] Li Y, Lu W, Schwartz AL, Bu G (2004) Degradation of the LDL receptor class 2 mutants is mediated by a proteasome-dependent pathway. J Lipid Res 45:1084–1091 10.1194/jlr.M300482-JLR200 14993243

[CR50] Liu Y, Zhang X, Liu J, Xia H, Zou J, Muruato AE, Periasamy S, Kurhade C, Plante JA, Bopp NE et al (2022) A live-attenuated SARS-CoV-2 vaccine candidate with accessory protein deletions. Nat Commun 13:4337 10.1038/s41467-022-31930-z PMC9326133 35896528

[CR51] Lo Surdo P, Bottomley MJ, Calzetta A, Settembre EC, Cirillo A, Pandit S, Ni YG, Hubbard B, Sitlani A, Carfi A (2011) Mechanistic implications for LDL receptor degradation from the PCSK9/LDLR structure at neutral pH. EMBO Rep 12:1300–1305 10.1038/embor.2011.205 PMC3245695 22081141

[CR52] Lu W, Zheng BJ, Xu K, Schwarz W, Du L, Wong CK, Chen J, Duan S, Deubel V, Sun B (2006) Severe acute respiratory syndrome-associated coronavirus 3a protein forms an ion channel and modulates virus release. Proc Natl Acad Sci USA 103:12540–12545 10.1073/pnas.0605402103 PMC1567914 16894145

[CR53] Lu Y, Liu DX, Tam JP (2008) Lipid rafts are involved in SARS-CoV entry into Vero E6 cells. Biochem Biophys Res Commun 369:344–349 10.1016/j.bbrc.2008.02.023 PMC7092920 18279660

[CR54] Maier HJ, Hawes PC, Cottam EM, Mantell J, Verkade P, Monaghan P, Wileman T, Britton P (2013) Infectious bronchitis virus generates spherules from zippered endoplasmic reticulum membranes. mBio 4:e00801–e00813 10.1128/mBio.00801-13 PMC3812713 24149513

[CR55] Ma-Lauer Y, Li P, Niemeyer D, Richter A, Pusl K, von Brunn B, Ru Y, Xiang C, Schwinghammer S, Liu J et al (2024) Oxysterole-binding protein targeted by SARS-CoV-2 viral proteins regulates coronavirus replication. Front Cell Infect Microbiol 14:1383917 10.3389/fcimb.2024.1383917 PMC11306179 39119292

[CR56] Maxwell KN, Fisher EA, Breslow JL (2005) Overexpression of PCSK9 accelerates the degradation of the LDLR in a post-endoplasmic reticulum compartment. Proc Natl Acad Sci USA 102:2069–2074 10.1073/pnas.0409736102 PMC546019 15677715

[CR57] McGrath ME, Xue Y, Dillen C, Oldfield L, Assad-Garcia N, Zaveri J, Singh N, Baracco L, Taylor LJ, Vashee S et al (2022) SARS-CoV-2 variant spike and accessory gene mutations alter pathogenesis. Proc Natl Acad Sci USA 119:e2204717119 10.1073/pnas.2204717119 PMC9477415 36040867

[CR58] Miao G, Zhao H, Li Y, Ji M, Chen Y, Shi Y, Bi Y, Wang P, Zhang H (2021) ORF3a of the COVID-19 virus SARS-CoV-2 blocks HOPS complex-mediated assembly of the SNARE complex required for autolysosome formation. Dev Cell 56:427–442.e425 10.1016/j.devcel.2020.12.010 PMC7832235 33422265

[CR59] Michelucci A, Sforna L, Focaia R, Leonardi MV, Di Battista A, Rastelli G, Vespa S, Boncompagni S, Di Cristina M, Catacuzzeno L (2025) SARS-CoV-2 ORF3a accessory protein is a water-permeable channel that induces lysosome swelling. Commun Biol 8:170 10.1038/s42003-024-07442-5 PMC11794868 39905220

[CR60] Miller AN, Houlihan PR, Matamala E, Cabezas-Bratesco D, Lee GY, Cristofori-Armstrong B, Dilan TL, Sanchez-Martinez S, Matthies D, Yan R et al (2023) The SARS-CoV-2 accessory protein Orf3a is not an ion channel, but does interact with trafficking proteins. eLife 12:e84477 10.7554/eLife.84477 PMC9910834 36695574

[CR61] Nagy A, Pongor S, Gyorffy B (2021) Different mutations in SARS-CoV-2 associate with severe and mild outcome. Int J Antimicrob Agents 57:106272 10.1016/j.ijantimicag.2020.106272 PMC7755579 33347989

[CR62] Nistor A, Simionescu M (1986) Uptake of low density lipoproteins by the hamster lung. Interactions with capillary endothelium. Am Rev Respir Dis 134:1266–1272 10.1164/arrd.1986.134.6.1266 3024539

[CR63] Omasits U, Ahrens CH, Muller S, Wollscheid B (2014) Protter: interactive protein feature visualization and integration with experimental proteomic data. Bioinformatics 30:884–886 10.1093/bioinformatics/btt607 24162465

[CR64] Oostra M, de Haan CA, de Groot RJ, Rottier PJ (2006) Glycosylation of the severe acute respiratory syndrome coronavirus triple-spanning membrane proteins 3a and M. J Virol 80:2326–2336 10.1128/JVI.80.5.2326-2336.2006 PMC1395384 16474139

[CR65] Ostendorf BN, Patel MA, Bilanovic J, Hoffmann HH, Carrasco SE, Rice CM, Tavazoie SF (2022) Common human genetic variants of APOE impact murine COVID-19 mortality. Nature 611:346–351 10.1038/s41586-022-05344-2 PMC10957240 36130725

[CR66] Petersen DN, Hawkins J, Ruangsiriluk W, Stevens KA, Maguire BA, O’Connell TN, Rocke BN, Boehm M, Ruggeri RB, Rolph T et al (2016) A small-molecule anti-secretagogue of PCSK9 targets the 80S ribosome to inhibit PCSK9 protein translation. Cell Chem Biol 23:1362–1371 10.1016/j.chembiol.2016.08.016 27746128

[CR67] Port JR, Adney DR, Schwarz B, Schulz JE, Sturdevant DE, Smith BJ, Avanzato VA, Holbrook MG, Purushotham JN, Stromberg KA et al (2021) High-fat high-sugar diet-induced changes in the lipid metabolism are associated with mildly increased COVID-19 severity and delayed recovery in the Syrian hamster. Viruses 13:2506 10.3390/v13122506 PMC8703573 34960775

[CR68] Radvak P, Kwon HJ, Kosikova M, Ortega-Rodriguez U, Xiang R, Phue JN, Shen RF, Rozzelle J, Kapoor N, Rabara T et al (2021) SARS-CoV-2 B.1.1.7 (alpha) and B.1.351 (beta) variants induce pathogenic patterns in K18-hACE2 transgenic mice distinct from early strains. Nat Commun 12:6559 10.1038/s41467-021-26803-w PMC8589842 34772941

[CR69] Rajah MM, Hubert M, Bishop E, Saunders N, Robinot R, Grzelak L, Planas D, Dufloo J, Gellenoncourt S, Bongers A et al (2021) SARS-CoV-2 alpha, beta, and delta variants display enhanced Spike-mediated syncytia formation. EMBO J 40:e108944 10.15252/embj.2021108944 PMC8646911 34601723

[CR70] Sachs N, Papaspyropoulos A, Zomer-van Ommen DD, Heo I, Bottinger L, Klay D, Weeber F, Huelsz-Prince G, Iakobachvili N, Amatngalim GD et al (2019) Long-term expanding human airway organoids for disease modeling. EMBO J 38:e100300 10.15252/embj.2018100300 PMC6376275 30643021

[CR71] Safdari Lord J, Soltani Rezaiezadeh J, Yekaninejad MS, Izadi P (2022) The association of APOE genotype with COVID-19 disease severity. Sci Rep 12:13483 10.1038/s41598-022-17262-4 PMC9356041 35931737

[CR72] Sanders DW, Jumper CC, Ackerman PJ, Bracha D, Donlic A, Kim H, Kenney D, Castello-Serrano I, Suzuki S, Tamura T et al (2021) SARS-CoV-2 requires cholesterol for viral entry and pathological syncytia formation. eLife 10:e65962 10.7554/eLife.65962 PMC8104966 33890572

[CR73] Schneider WM, Luna JM, Hoffmann HH, Sanchez-Rivera FJ, Leal AA, Ashbrook AW, Le Pen J, Ricardo-Lax I, Michailidis E, Peace A et al (2021) Genome-scale identification of SARS-CoV-2 and pan-coronavirus host factor networks. Cell 184:120–132.e114 10.1016/j.cell.2020.12.006 PMC7796900 33382968

[CR74] Snijder EJ, Limpens R, de Wilde AH, de Jong AWM, Zevenhoven-Dobbe JC, Maier HJ, Faas F, Koster AJ, Barcena M (2020) A unifying structural and functional model of the coronavirus replication organelle: tracking down RNA synthesis. PLoS Biol 18:e3000715 10.1371/journal.pbio.3000715 PMC7302735 32511245

[CR75] Solvik TA, Nguyen TA, Tony Lin YH, Marsh T, Huang EJ, Wiita AP, Debnath J, Leidal AM (2022) Secretory autophagy maintains proteostasis upon lysosome inhibition. J Cell Biol 221:e202110151 10.1083/jcb.202110151 PMC9036093 35446347

[CR76] Stukalov A, Girault V, Grass V, Karayel O, Bergant V, Urban C, Haas DA, Huang Y, Oubraham L, Wang A et al (2021) Multilevel proteomics reveals host perturbations by SARS-CoV-2 and SARS-CoV. Nature 594:246–252 10.1038/s41586-021-03493-4 33845483

[CR77] Su YCF, Anderson DE, Young BE, Linster M, Zhu F, Jayakumar J, Zhuang Y, Kalimuddin S, Low JGH, Tan CW et al (2020) Discovery and genomic characterization of a 382-nucleotide deletion in ORF7b and ORF8 during the early evolution of SARS-CoV-2. mBio 11:e01610–e01620 10.1128/mBio.01610-20 PMC7374062 32694143

[CR78] Syed AM, Taha TY, Tabata T, Chen IP, Ciling A, Khalid MM, Sreekumar B, Chen PY, Hayashi JM, Soczek KM et al (2021) Rapid assessment of SARS-CoV-2-evolved variants using virus-like particles. Science 374:1626–1632 10.1126/science.abl6184 PMC9005165 34735219

[CR79] Taha TY, Chen IP, Hayashi JM, Tabata T, Walcott K, Kimmerly GR, Syed AM, Ciling A, Suryawanshi RK, Martin HS et al (2023) Rapid assembly of SARS-CoV-2 genomes reveals attenuation of the Omicron BA.1 variant through NSP6. Nat Commun 14:2308 10.1038/s41467-023-37787-0 PMC10120482 37085489

[CR80] Tamara A, Tahapary DL (2020) Obesity as a predictor for a poor prognosis of COVID-19: a systematic review. Diab Metab Syndr 14:655–659 10.1016/j.dsx.2020.05.020 PMC7217103 32438328

[CR81] Tan WYT, Young BE, Lye DC, Chew DEK, Dalan R (2020) Statin use is associated with lower disease severity in COVID-19 infection. Sci Rep 10:17458 10.1038/s41598-020-74492-0 PMC7562925 33060704

[CR82] Tarres-Freixas F, Trinite B, Pons-Grifols A, Romero-Durana M, Riveira-Munoz E, Avila-Nieto C, Perez M, Garcia-Vidal E, Perez-Zsolt D, Munoz-Basagoiti J et al (2022) Heterogeneous infectivity and pathogenesis of SARS-CoV-2 variants beta, delta and omicron in transgenic K18-hACE2 and wildtype mice. Front Microbiol 13:840757 10.3389/fmicb.2022.840757 PMC9114491 35602059

[CR83] Trimarco JD, Heaton BE, Chaparian RR, Burke KN, Binder RA, Gray GC, Smith CM, Menachery VD, Heaton NS (2021) TMEM41B is a host factor required for the replication of diverse coronaviruses including SARS-CoV-2. PLoS Pathog 17:e1009599 10.1371/journal.ppat.1009599 PMC8189496 34043740

[CR84] Turley SD, Andersen JM, Dietschy JM (1981) Rates of sterol synthesis and uptake in the major organs of the rat in vivo. J Lipid Res 22:551–569 7276735

[CR85] Tveten K, Str MT, Berge KE, Leren TP (2013) PCSK9-mediated degradation of the LDL receptor generates a 17 kDa C-terminal LDL receptor fragment. J Lipid Res 54:1560–1566 10.1194/jlr.M034371 PMC3646457 23509406

[CR86] Vos DY, van de Sluis B (2021) Function of the endolysosomal network in cholesterol homeostasis and metabolic-associated fatty liver disease (MAFLD). Mol Metab 50:101146 10.1016/j.molmet.2020.101146 PMC8324686 33348067

[CR87] Walia K, Sharma A, Paul S, Chouhan P, Kumar G, Ringe R, Sharma M, Tuli A (2024) SARS-CoV-2 virulence factor ORF3a blocks lysosome function by modulating TBC1D5-dependent Rab7 GTPase cycle. Nat Commun 15:2053 10.1038/s41467-024-46417-2 PMC10918171 38448435

[CR88] Wang H, Yuan Z, Pavel MA, Jablonski SM, Jablonski J, Hobson R, Valente S, Reddy CB, Hansen SB (2023) The role of high cholesterol in SARS-CoV-2 infectivity. J Biol Chem 299:104763 10.1016/j.jbc.2023.104763 PMC10140059 37119851

[CR89] Wang R, Simoneau CR, Kulsuptrakul J, Bouhaddou M, Travisano KA, Hayashi JM, Carlson-Stevermer J, Zengel JR, Richards CM, Fozouni P et al (2021a) Genetic screens identify host factors for SARS-CoV-2 and common cold coronaviruses. Cell 184:106–119.e114 10.1016/j.cell.2020.12.004 PMC7723770 33333024

[CR90] Wang R, Yang X, Chang M, Xue Z, Wang W, Bai L, Zhao S, Liu E (2021b) ORF3a protein of severe acute respiratory syndrome coronavirus 2 inhibits interferon-activated janus kinase/signal transducer and activator of transcription signaling via elevating suppressor of cytokine signaling 1. Front Microbiol 12:752597 10.3389/fmicb.2021.752597 PMC8506155 34650546

[CR91] Wang S, Li W, Hui H, Tiwari SK, Zhang Q, Croker BA, Rawlings S, Smith D, Carlin AF, Rana TM (2020) Cholesterol 25-hydroxylase inhibits SARS-CoV-2 and other coronaviruses by depleting membrane cholesterol. EMBO J 39:e106057 10.15252/embj.2020106057 PMC7537045 32944968

[CR92] Wei J, Alfajaro MM, DeWeirdt PC, Hanna RE, Lu-Culligan WJ, Cai WL, Strine MS, Zhang SM, Graziano VR, Schmitz CO et al (2021) Genome-wide CRISPR screens reveal host factors critical for SARS-CoV-2 infection. Cell 184:76–91.e13 10.1016/j.cell.2020.10.028 PMC7574718 33147444

[CR93] Xu H, Akinyemi IA, Chitre SA, Loeb JC, Lednicky JA, McIntosh MT, Bhaduri-McIntosh S (2022) SARS-CoV-2 viroporin encoded by ORF3a triggers the NLRP3 inflammatory pathway. Virology 568:13–22 10.1016/j.virol.2022.01.003 PMC8762580 35066302

[CR94] Yu CJ, Chen YC, Hsiao CH, Kuo TC, Chang SC, Lu CY, Wei WC, Lee CH, Huang LM, Chang MF et al (2004) Identification of a novel protein 3a from severe acute respiratory syndrome coronavirus. FEBS Lett 565:111–116 10.1016/j.febslet.2004.03.086 PMC7126674 15135062

[CR95] Zang R, Case JB, Yutuc E, Ma X, Shen S, Gomez Castro MF, Liu Z, Zeng Q, Zhao H, Son J et al (2020) Cholesterol 25-hydroxylase suppresses SARS-CoV-2 replication by blocking membrane fusion. Proc Natl Acad Sci USA 117:32105–32113 10.1073/pnas.2012197117 PMC7749331 33239446

[CR96] Zhang J, Ejikemeuwa A, Gerzanich V, Nasr M, Tang Q, Simard JM, Zhao RY (2022a) Understanding the role of SARS-CoV-2 ORF3a in viral pathogenesis and COVID-19. Front Microbiol 13:854567 10.3389/fmicb.2022.854567 PMC8959714 35356515

[CR97] Zhang S, Nelson OD, Price IR, Zhu C, Lu X, Fernandez IR, Weiss RS, Lin H (2022b) Long-chain fatty acyl coenzyme A inhibits NME1/2 and regulates cancer metastasis. Proc Natl Acad Sci USA 119:e2117013119 10.1073/pnas.2117013119 PMC8931372 35259022

[CR98] Zhang X, Liu Y, Liu J, Bailey AL, Plante KS, Plante JA, Zou J, Xia H, Bopp NE, Aguilar PV et al (2021) A trans-complementation system for SARS-CoV-2 recapitulates authentic viral replication without virulence. Cell 184:2229–2238.e2213 10.1016/j.cell.2021.02.044 PMC7901297 33691138

[CR99] Zhang XJ, Qin JJ, Cheng X, Shen L, Zhao YC, Yuan Y, Lei F, Chen MM, Yang H, Bai L et al (2020) In-hospital use of statins is associated with a reduced risk of mortality among individuals with COVID-19. Cell Metab 32:176–187.e174 10.1016/j.cmet.2020.06.015 PMC7311917 32592657

[CR100] Zhou J, Li C, Sachs N, Chiu MC, Wong BH, Chu H, Poon VK, Wang D, Zhao X, Wen L et al (2018) Differentiated human airway organoids to assess infectivity of emerging influenza virus. Proc Natl Acad Sci USA 115:6822–6827 10.1073/pnas.1806308115 PMC6042130 29891677

