# Supplementary material for: Circulating cholesterol fuels SARS-CoV-2 replication via ORF3a
Source: EMBO Rep. 2026 Sep 11;27(20):5991–6017. doi: 10.1038/s44319-026-00907-0 (PMC13694717; doi:10.1038/s44319-026-00907-0)
Supplement: Supplementary file 2 [file 44319_2026_907_MOESM6_ESM.pdf]

Expanded View Figures

Figure EV1. SARS-CoV-2 infection of human airway cells is enhanced by extracellular LDL.

(**A**) Dose-dependent increase in SARS-CoV-2 infection in A549-ACE2 cells upon LDL treatment, measured by quantifying dsRNA-positive cells via automated imaging. $N = 3$ biological replicates. One-way ANOVA. (**B**) Infectious virion production from A549-ACE2 cells following LDL treatment measured by plaque forming units (PFU) in Vero E6 cells. $N = 3$ biological replicates. Paired *t* test. (**C**) Quantification of dsRNA-positive A549-ACE2 cells infected with SARS-CoV-2 WA1 in the presence or absence of VLDL. $N = 3$ biological replicates. Unpaired *t* test. (**D**) Representative Western blot of LDLR and GAPDH expression in A549-ACE2 cells treated with increasing doses of (R)-IMPP for 24 h. (**E**) Quantification of dsRNA-positive A549-ACE2 cells treated with 40 μM cholesterol complexed with methyl-beta-cyclodextrin. $N = 3$ biological replicates. Unpaired *t* test. (**F**) Quantification of mean fluorescent intensity (MFI) of LipidTOX Deep Red (LDR) in A549-ACE2 cells infected with WA1 or Beta variant SARS-CoV-2, with or without LDL treatment. MOI 0.1 for 72 h. $N = 3$ biological replicates. Unpaired *t* test. (**G**) Beta variant infectious virion production from A549-ACE2 cells upon LDL treatment measured by PFU in Vero E6 cells. $N = 3$ biological replicates. Unpaired *t* test. (**H**) Copies of viral RNA (cellular and supernatant) measured by RT-qPCR against the viral N gene, and infectious virion production measured by PFU in A549-ACE2 cells over 4 days. $N = 3$ biological replicates. Unpaired *t* test. Data information: In (**A–C**, **E–H**), data are presented as mean ± SEM. ***$P < 0.001$, **$P < 0.01$, *$P < 0.05$.

© The Author(s)

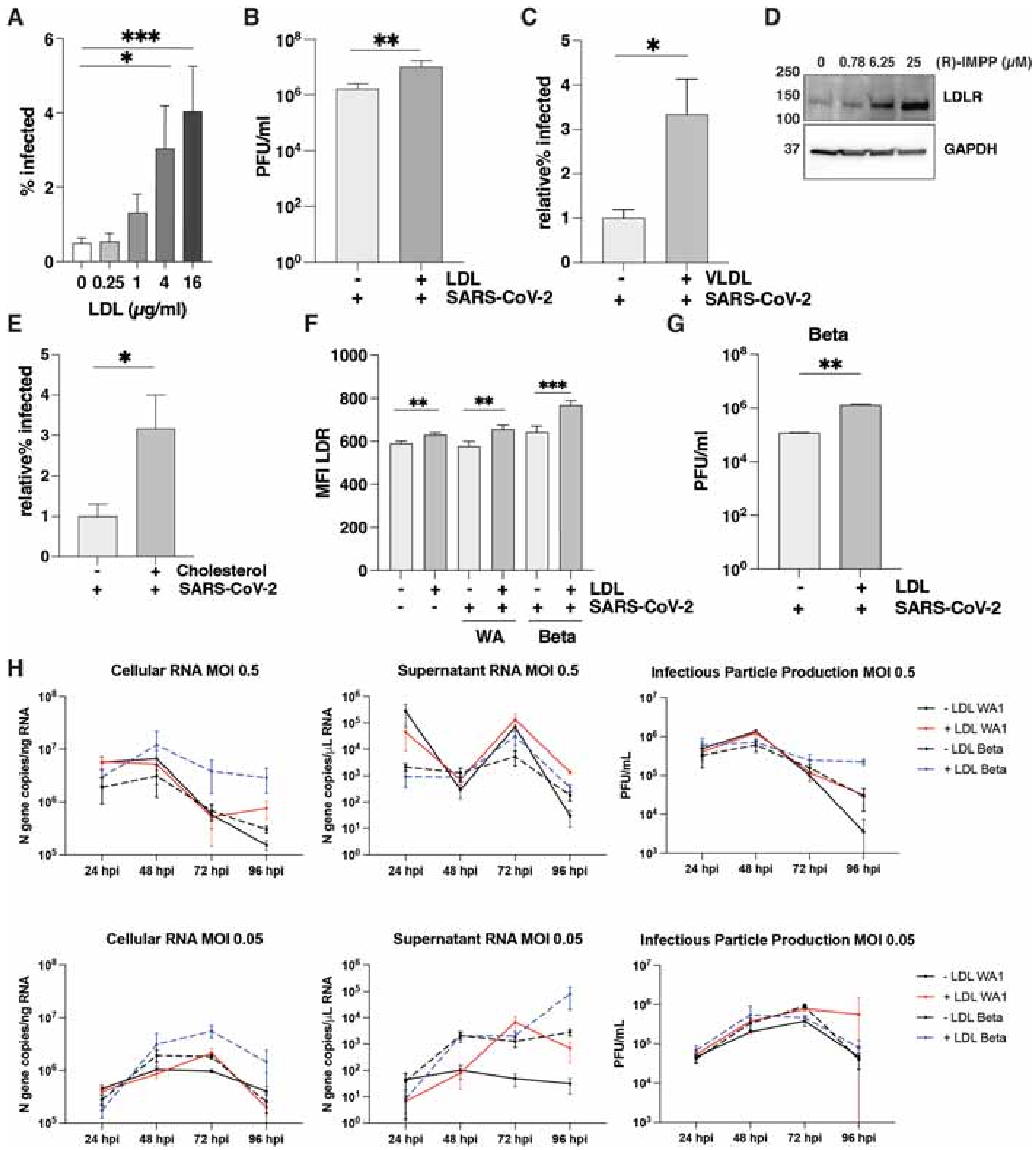
A
% infected
LDL (μg/ml)
B
PFU/ml
LDL
SARS-CoV-2
C
relative% infected
VLDL
SARS-CoV-2
D
(R)-IMPP (μM)
LDLR
GAPDH
E
relative% infected
Cholesterol
SARS-CoV-2
F
MFI LDR
LDL
SARS-CoV-2
WA
Beta
G
Beta
PFU/ml
LDL
SARS-CoV-2
H
Cellular RNA MOI 0.5
Supernatant RNA MOI 0.5
Infectious Particle Production MOI 0.5
Cellular RNA MOI 0.05
Supernatant RNA MOI 0.05
Infectious Particle Production MOI 0.05
N gene copies/ng RNA
N gene copies/μL RNA
PFU/mL
- LDL WA1
+ LDL WA1
- LDL Beta
+ LDL Beta

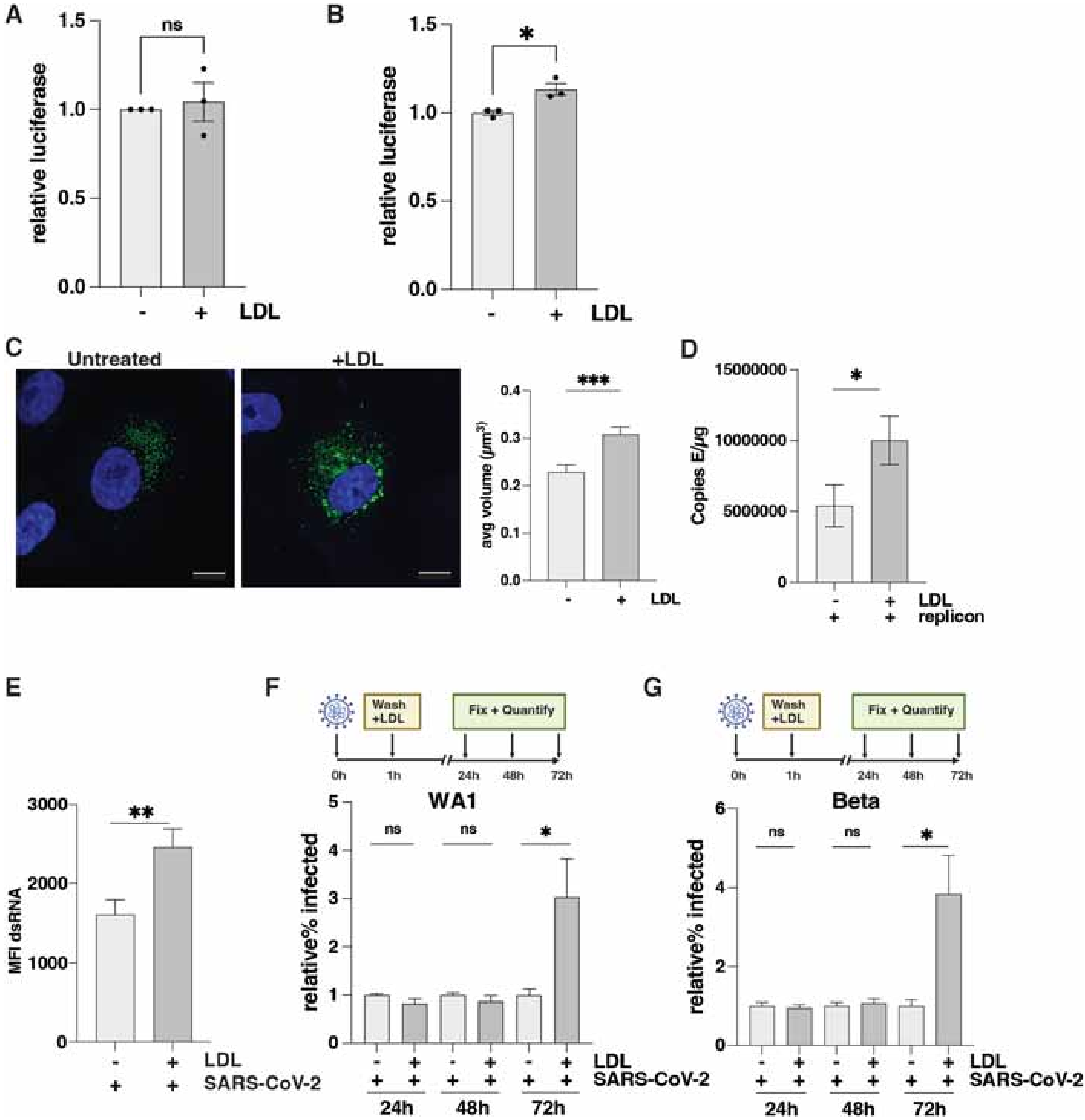

Figure EV2. LDL treatment increases viral dsRNA-positive complexes.

(**A**) Luminescence analysis of A549-ACE2 cells transduced with Spike-carrying pseudotyped lentiviral vectors with or without LDL. $N = 3$ biological replicates. Unpaired *t* test. (**B**) Luminescence analysis of A549-ACE2 cells infected with single-round SARS-CoV-2 WA1 replicon in the presence or absence of LDL. $N = 3$ biological replicates. Unpaired *t* test. (**C**) Representative images and quantification of dsRNA complex volume in Vero E6-ACE2/TMPRSS2 cells infected with single-round SARS-CoV-2 WA1 replicon virus in the presence or absence of LDL, stained for dsRNA (green) replication complex volume. Scale bar = 10 μm. $N = 3$ biological replicates. Unpaired *t* test. (**D**) RT-qPCR analysis of viral E gene from cellular RNA in A549-ACE2 cells infected with single-round replicon of SARS-CoV-2 WA1 in the presence or absence of LDL. $N = 3$ biological replicates. Unpaired *t* test. (**E**) Quantification of dsRNA fluorescence mean intensity (MFI) in A549-ACE2 cells infected with SARS-CoV-2 WA1 for 1 h, washed and replaced with media with or without LDL. $N = 3$ biological replicates. Unpaired *t* test. Quantification of dsRNA-positive A549-ACE2 cells infected with SARS-CoV-2 WA1 (**F**) or SARS-CoV-2 Beta (**G**) for 1 h, washed, and replaced with media with or without LDL at different time points. $N = 3$ biological replicates. Unpaired *t* test. Data information: In (**A–D**), data are presented as mean ± SEM. ***$P < 0.001$, **$P < 0.01$, *$P < 0.05$.

© The Author(s)

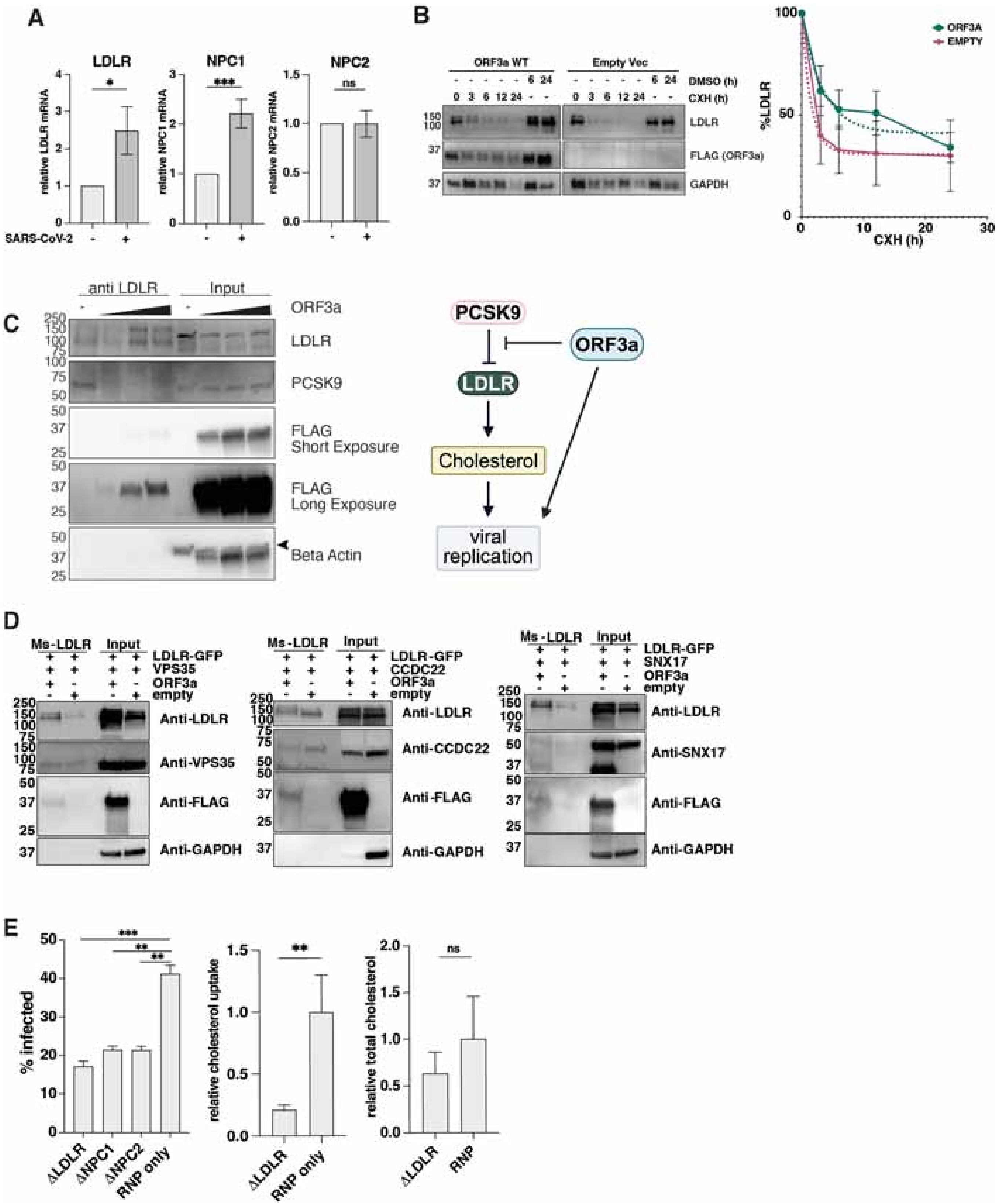
A
LDLR
NPC1
NPC2
relative LDLR mRNA
relative NPC1 mRNA
relative NPC2 mRNA
SARS-CoV-2
ns
B
ORF3a WT
Empty Vec
DMSO (h)
CXH (h)
LDLR
FLAG (ORF3a)
GAPDH
%LDLR
CXH (h)
ORF3A
EMPTY
C
anti LDLR
Input
ORF3a
LDLR
PCSK9
FLAG
Short Exposure
FLAG
Long Exposure
Beta Actin
PCSK9
ORF3a
LDLR
Cholesterol
viral
replication
D
Ms-LDLR
Input
LDLR-GFP
VPS35
ORF3a
empty
Anti-LDLR
Anti-VPS35
Anti-FLAG
Anti-GAPDH
CCDC22
Anti-CCDC22
SNX17
Anti-SNX17
E
% infected
ΔLDLR
ΔNPC1
ΔNPC2
RNP only
relative cholesterol uptake
relative total cholesterol
RNP

Figure EV3. SARS-CoV-2 requires cholesterol uptake genes.

(**A**) RT-qPCR of cellular RNA from A549-ACE2 cells infected with SARS-CoV-2 WA1 using primers against human *ldlr*, *npc1*, and *npc2*. $N = 3$ biological replicates. Unpaired *t* test. (**B**) Representative Western blots and quantification of cycloheximide (CXH) or DMSO-treated HEK293T cells expressing ORF3a-FLAG and GFP-LDLR over time. $N = 3$ biological replicates. Dotted lines indicate nonlinear regression best-fit curves. $R^2 = 0.7047$ (ORF3a) and 0.6684 (empty vector control). (**C**) Model and representative Western blot of anti-LDLR co-immunoprecipitation of LDLR, PCSK9, ORF3a-FLAG, and β-actin in HEK293T cells expressing ORF3a-FLAG. Arrowhead indicate expected band for β-actin. (**D**) Representative western blot of anti-LDLR co-immunoprecipitation of LDLR, VPS35, CCDC22, SNX17, ORF3a-FLAG, and GAPDH in HEK293T cells overexpressing GFP-LDLR, ORF3a-FLAG, and respective CCC/WASH/HOPS complex members. (**E**) Left panel, Quantification of dsRNA-positive A549-ACE2 cells infected with SARS-CoV-2 WA1 in CRISPR knockout lines (ΔLDLR, ΔNPC1, or ΔNPC2) or RNP only controls. $N = 3$ biological replicates. Unpaired *t* test. Middle panel, DiI-LDL uptake by mean fluorescence intensity in ΔLDLR or RNP only control A549-ACE2 cells. $N = 3$ biological replicates. Unpaired *t* test. Right panel, total intracellular cholesterol using Filipin III staining (mean fluorescence intensity) compared to RNP control in ΔLDLR or RNP only A549-ACE2 cells. $N = 3$ biological replicates. Unpaired *t* test. Infections performed at an MOI of 0.1 for 72 h. Data information: In (**A**, **B**, **E**), data are presented as mean ± SEM. ***$P < 0.001$, **$P < 0.01$, *$P < 0.05$.

 © The Author(s)

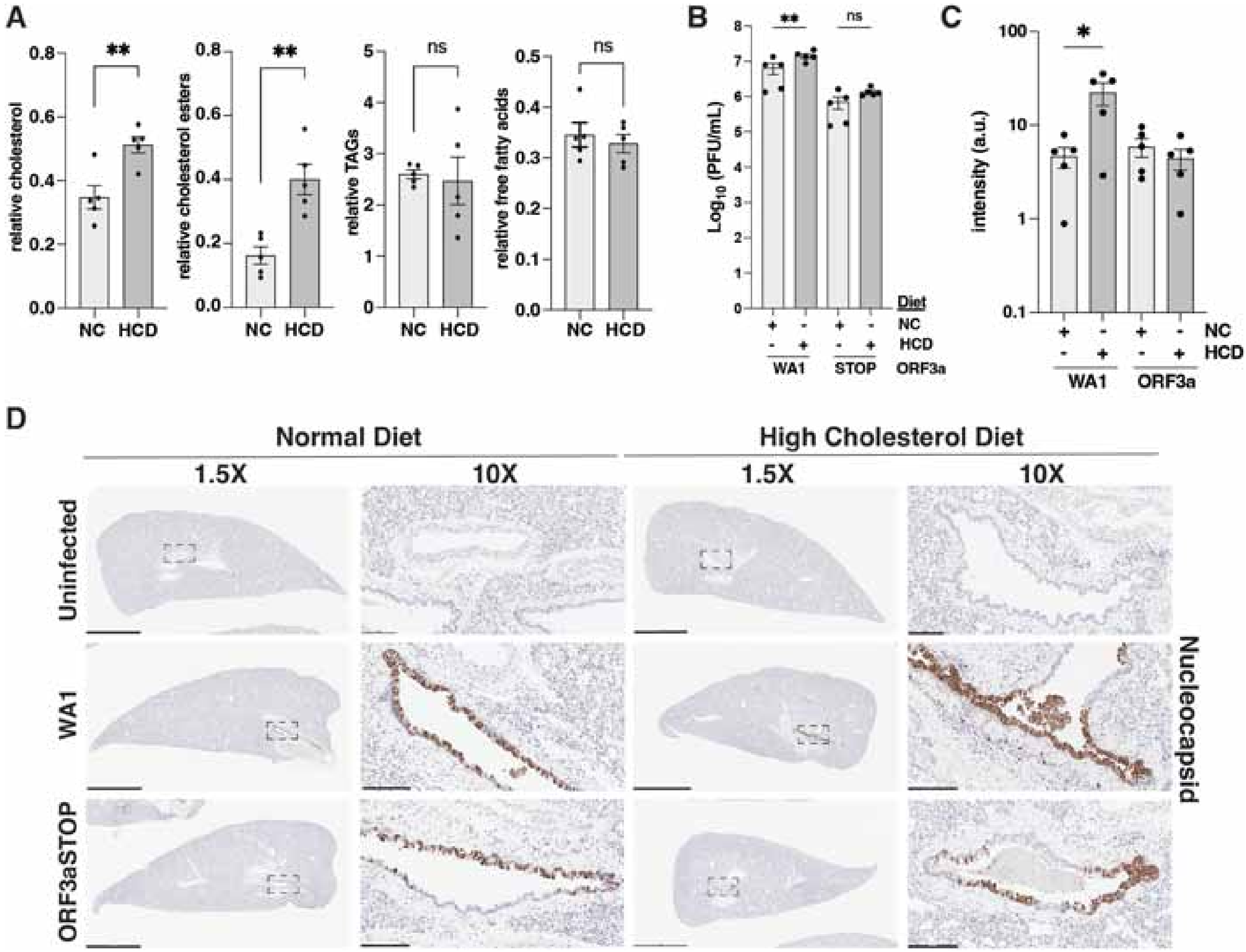

Figure EV4. SARS-CoV-2 infection is enhanced in vivo with a high cholesterol diet.

(**A**) Quantification of relative spot intensities of cholesterol, cholesterol esters, triglycerides, and free fatty acids from TLCs (normalized to 100 µg lipid standards) from uninfected lungs of Golden Syrian hamsters fed normal chow (NC) or high cholesterol diet (HCD). $N = 5$ animals per group. One-way ANOVA. (**B**) Infectious virion production in upper airway tissue from infected NC- or HCD-fed Golden Syrian Hamsters. $N = 5$ animals per group. One-way ANOVA. (**C**) Quantification of SARS-CoV-2 nucleocapsid staining in lungs by immunohistochemistry, shown as integrated density. $N = 5$ animals per group. One-way ANOVA. (**D**) Representative immunohistochemistry images of uninfected, WA1-infected, and ORF3a-STOP-infected hamster lungs, stained against viral N protein at ×1.5 zoom (left panel, scale bar = 2 mm) and 10X zoom (right panel, scale bar = 200 µm), fed either NC or HCD. Data information: In (**A**–**C**), data are presented as mean ± SEM. $^{**}P < 0.01$, $^{*}P < 0.05$.

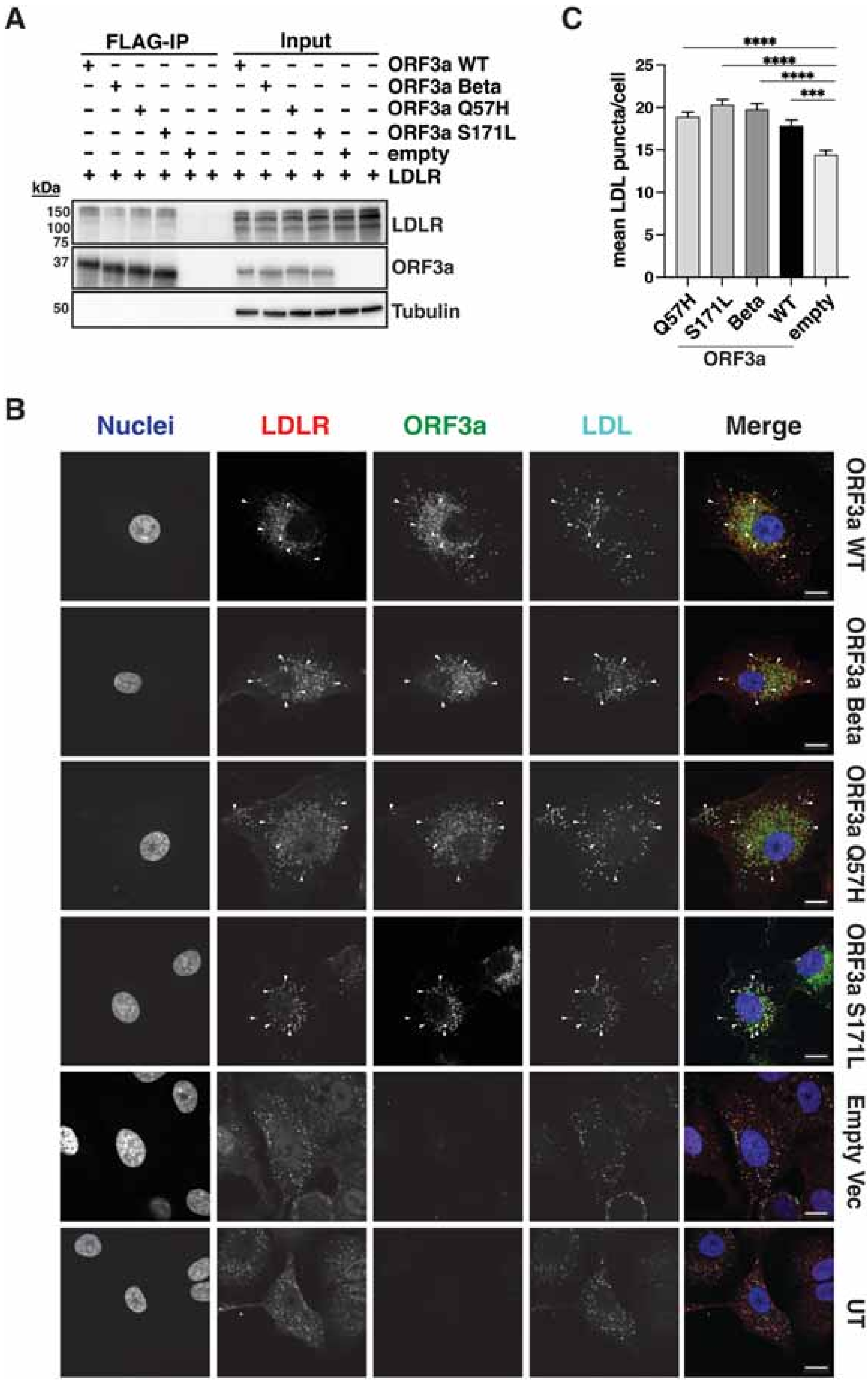
A
FLAG-IP
Input
ORF3a WT
ORF3a Beta
ORF3a Q57H
ORF3a S171L
empty
LDLR
kDa
150
100
75
37
50
LDLR
ORF3a
Tubulin
C
mean LDL puncta/cell
25
20
15
10
5
0
****
****
****
***
Q57H
S171L
Beta
WT
empty
ORF3a
B
Nuclei
LDLR
ORF3a
LDL
Merge
ORF3a WT
ORF3a Beta
ORF3a Q57H
ORF3a S171L
Empty Vec
UT

Figure EV5. SARS-CoV-2 ORF3a interacts with host LDLR at endosomes.

(**A**) Representative Western blot of anti-FLAG co-immunoprecipitation of HEK293T cells overexpressing GFP-LDLR and WT or mutant ORF3a-FLAG constructs. (**B**) Representative confocal microscopy images of A549 cells overexpressing WT or mutant ORF3a-FLAG constructs (green), stained for endogenous LDLR (red), and treated with fluorescently labeled LDL (cyan). Arrowheads indicate puncta containing LDLR, LDL, and ORF3a. Scale bar = 10 µm. (**C**) Quantification of LDL-pHrodo Red puncta in A549 cells expressing mutant or WT ORF3a, or empty vector. $N = 3$ biological replicates. One-way ANOVA. Data information: In (**C**), data are presented as mean ± SEM. ****$P < 0.0001$, ***$P < 0.001$.

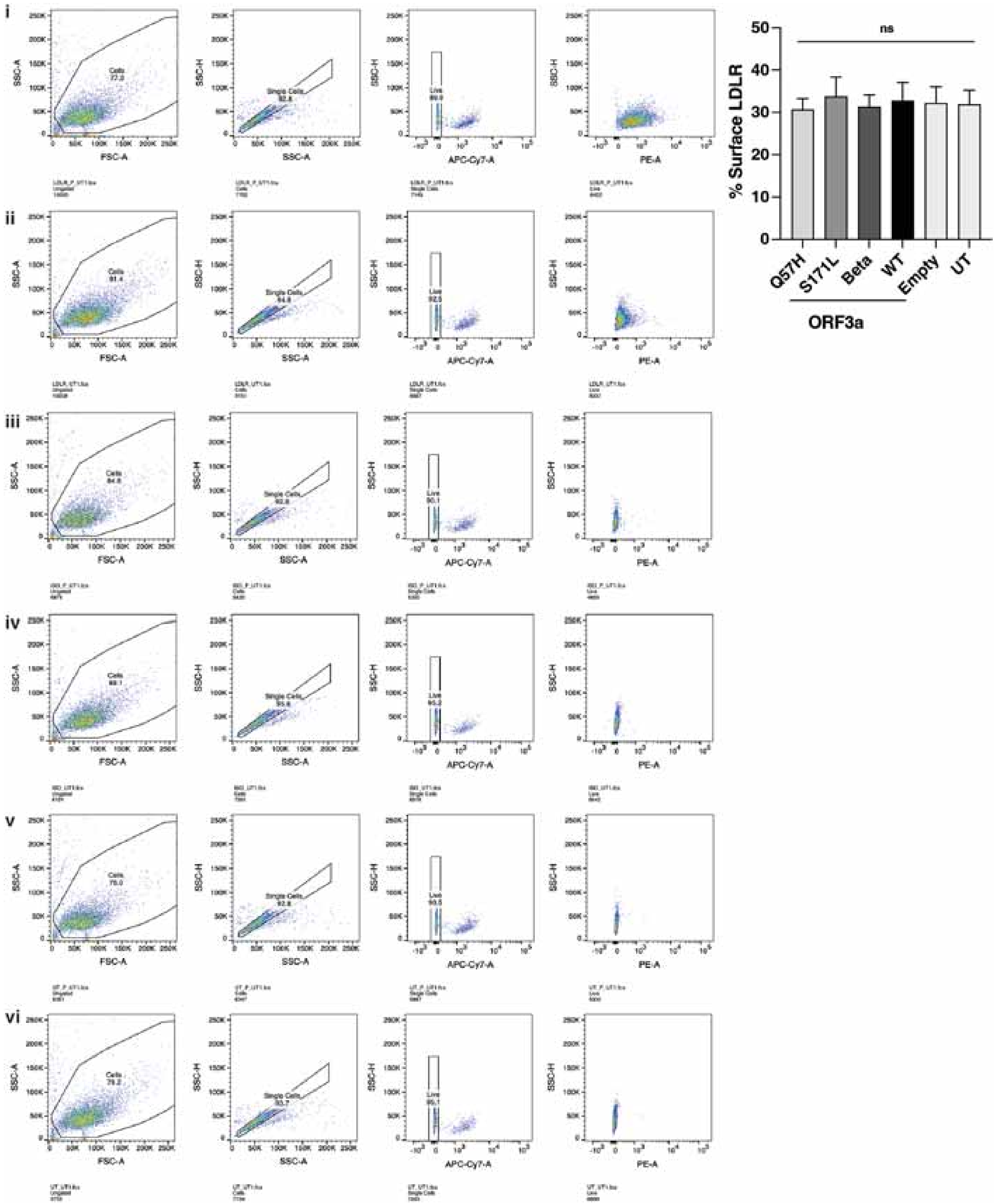
i
ii
iii
iv
v
vi
SSC-A
FSC-A
SSC-H
SSC-A
APC-Cy7-A
PE-A
Cells
Single Cells
Live
% Surface LDLR
ns
Q57H
S171L
Beta
WT
Empty
UT
ORF3a

Figure EV6. SARS-CoV-2 ORF3a does not significantly change LDLR surface presentation.

Flow cytometry gating strategy to measure total versus surface LDLR protein abundance in no antibody (UT) control A549-ACE2 cells. Cells were stained with eFlour 780 viability dye, and anti-LDLR-PE (LDLR) or isotype control-PE (ISO) antibodies in the presence (P) or absence of 0.1% saponin permeabilization. Quantification of surface (no detergent) versus total (saponin) LDLR in A549-ACE2 cells expressing empty vector, WT or mutant ORF3a. $N = 3$ biological replicates. One-way ANOVA. Data information: In (bar graph), data are presented as mean ± SEM.
