# Supplementary material for: Circulating cholesterol fuels SARS-CoV-2 replication via ORF3a
Source: EMBO Rep. 2026 Sep 11;27(20):5991–6017. doi: 10.1038/s44319-026-00907-0 (PMC13694717; doi:10.1038/s44319-026-00907-0)
Supplement: Supplementary file 5 [file 44319_2026_907_MOESM4_ESM.zip › Figure 3/3F/ORF3a-FLAG IP whole blot.pdf]

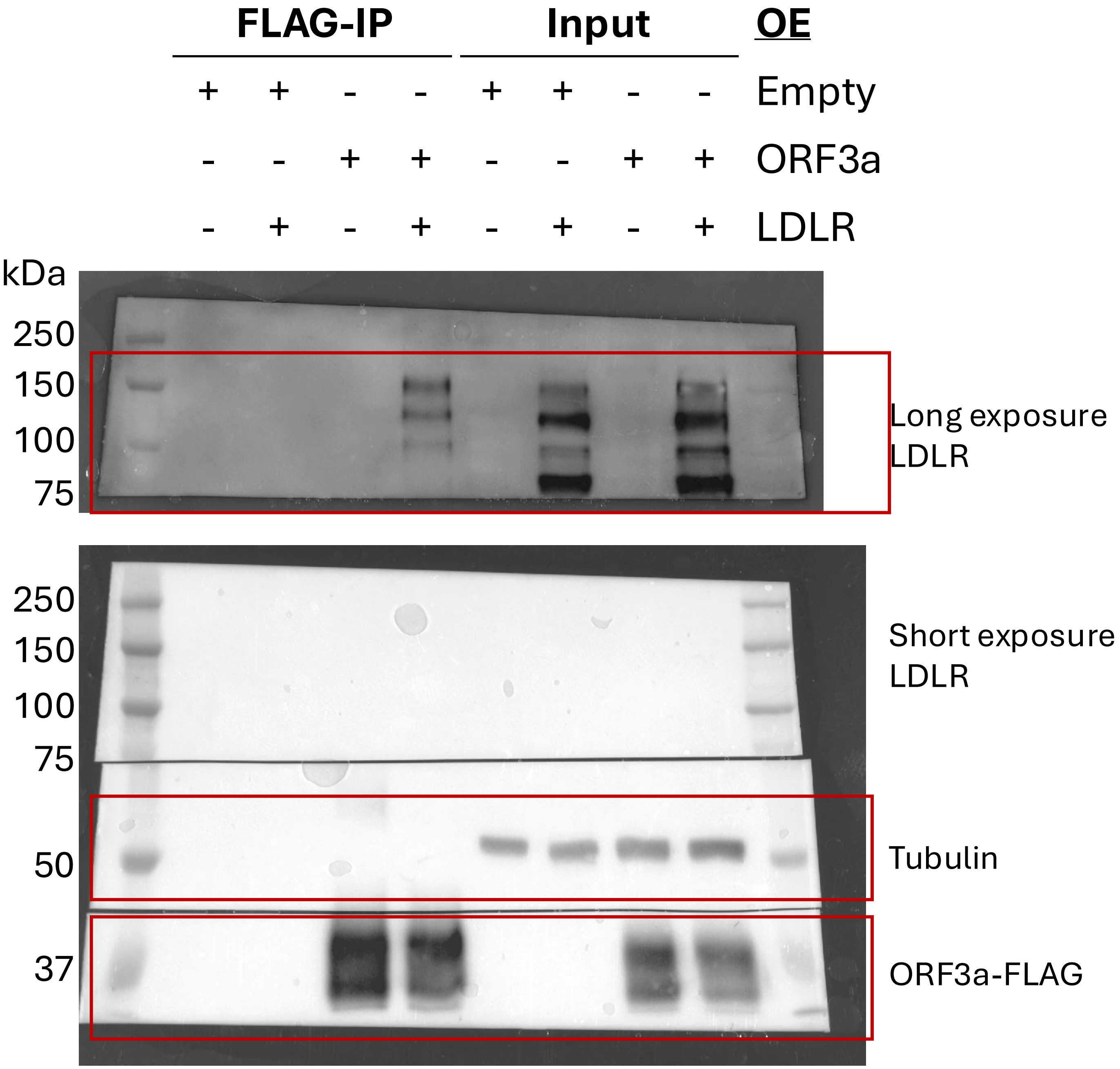

README: Blot was cut, individual sections stained for assigned proteins. Imaged together (lower panel). LDLR required reprobe and longer exposure alone (upper panel)
