# Supplementary material for: Circulating cholesterol fuels SARS-CoV-2 replication via ORF3a
Source: EMBO Rep. 2026 Sep 11;27(20):5991–6017. doi: 10.1038/s44319-026-00907-0 (PMC13694717; doi:10.1038/s44319-026-00907-0)
Supplement: Supplementary file 6 [file 44319_2026_907_MOESM5_ESM.zip › Figure 4/4D/ORF3a-FLAG IP Panels.pdf]

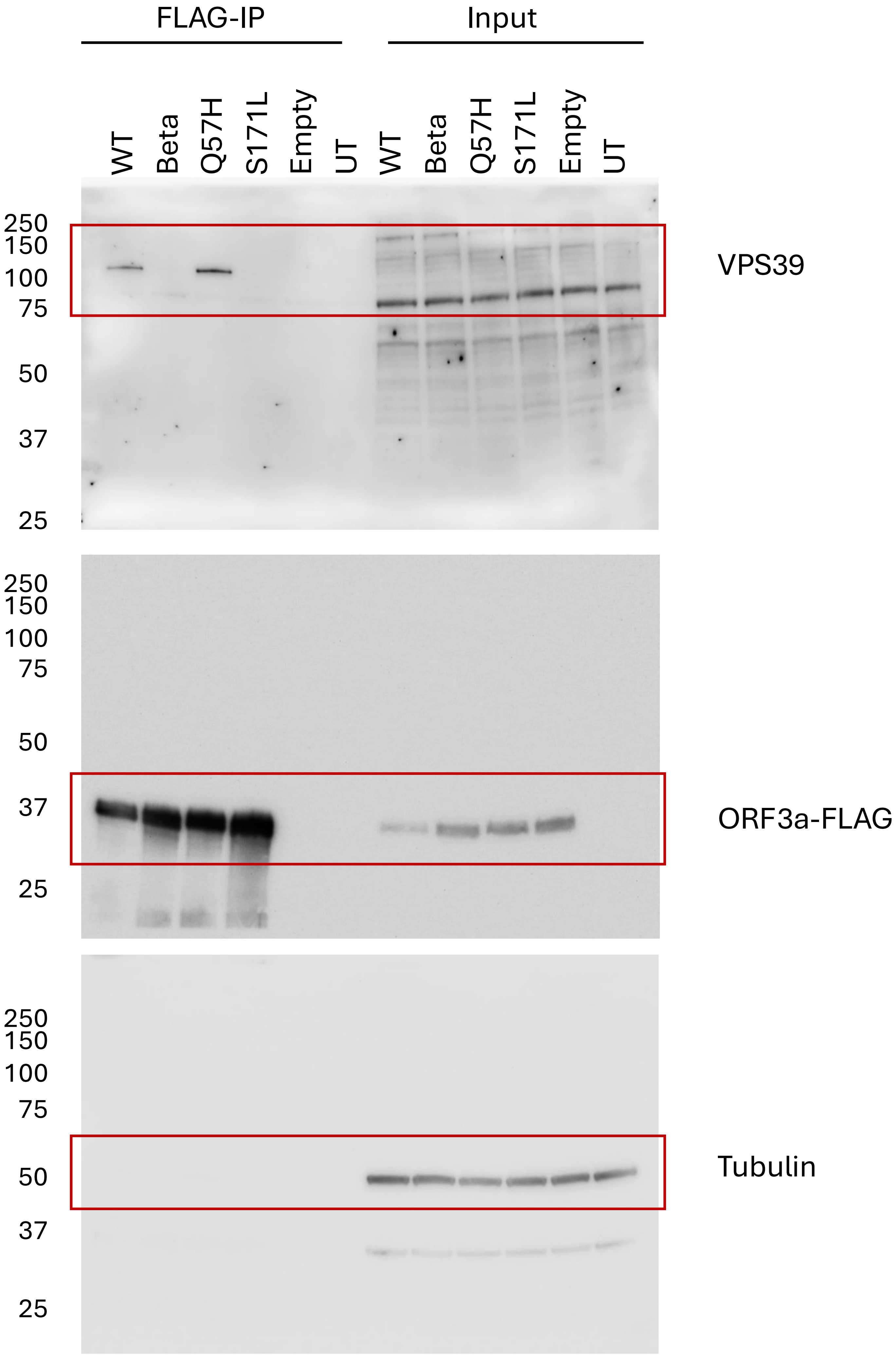
FLAG-IP
Input
WT
Beta
Q57H
S171L
Empty
UT
WT
Beta
Q57H
S171L
Empty
UT
250
150
100
75
50
37
25
VPS39
250
150
100
75
50
37
25
ORF3a-FLAG
250
150
100
75
50
37
25
Tubulin
